# Structural MRI predicts clinical progression in presymptomatic genetic frontotemporal dementia: findings from the GENetic Frontotemporal dementia Initiative cohort

**DOI:** 10.1093/braincomms/fcad061

**Published:** 2023-03-10

**Authors:** Martina Bocchetta, Emily G Todd, Arabella Bouzigues, David M Cash, Jennifer M Nicholas, Rhian S Convery, Lucy L Russell, David L Thomas, Ian B Malone, Juan Eugenio Iglesias, John C van Swieten, Lize C Jiskoot, Harro Seelaar, Barbara Borroni, Daniela Galimberti, Raquel Sanchez-Valle, Robert Laforce, Fermin Moreno, Matthis Synofzik, Caroline Graff, Mario Masellis, Maria Carmela Tartaglia, James B Rowe, Rik Vandenberghe, Elizabeth Finger, Fabrizio Tagliavini, Alexandre de Mendonça, Isabel Santana, Chris R Butler, Simon Ducharme, Alexander Gerhard, Adrian Danek, Johannes Levin, Markus Otto, Sandro Sorbi, Isabelle Le Ber, Florence Pasquier, Jonathan D Rohrer, Genetic Frontotemporal dementia Initiative (GENFI), Aitana Sogorb Esteve, Annabel Nelson, Carolin Heller, Caroline V Greaves, Hanya Benotmane, Henrik Zetterberg, Imogen J Swift, Kiran Samra, Rachelle Shafei, Carolyn Timberlake, Thomas Cope, Timothy Rittman, Alberto Benussi, Enrico Premi, Roberto Gasparotti, Silvana Archetti, Stefano Gazzina, Valentina Cantoni, Andrea Arighi, Chiara Fenoglio, Elio Scarpini, Giorgio Fumagalli, Vittoria Borracci, Giacomina Rossi, Giorgio Giaccone, Giuseppe Di Fede, Paola Caroppo, Pietro Tiraboschi, Sara Prioni, Veronica Redaelli, David Tang-Wai, Ekaterina Rogaeva, Miguel Castelo-Branco, Morris Freedman, Ron Keren, Sandra Black, Sara Mitchell, Christen Shoesmith, Robart Bartha, Rosa Rademakers, Jackie Poos, Janne M Papma, Lucia Giannini, Rick van Minkelen, Yolande Pijnenburg, Benedetta Nacmias, Camilla Ferrari, Cristina Polito, Gemma Lombardi, Valentina Bessi, Michele Veldsman, Christin Andersson, Hakan Thonberg, Linn Öijerstedt, Vesna Jelic, Paul Thompson, Tobias Langheinrich, Albert Lladó, Anna Antonell, Jaume Olives, Mircea Balasa, Nuria Bargalló, Sergi Borrego-Ecija, Ana Verdelho, Carolina Maruta, Catarina B Ferreira, Gabriel Miltenberger, Frederico Simões do Couto, Alazne Gabilondo, Ana Gorostidi, Jorge Villanua, Marta Cañada, Mikel Tainta, Miren Zulaica, Myriam Barandiaran, Patricia Alves, Benjamin Bender, Carlo Wilke, Lisa Graf, Annick Vogels, Mathieu Vandenbulcke, Philip Van Damme, Rose Bruffaerts, Koen Poesen, Pedro Rosa-Neto, Serge Gauthier, Agnès Camuzat, Alexis Brice, Anne Bertrand, Aurélie Funkiewiez, Daisy Rinaldi, Dario Saracino, Olivier Colliot, Sabrina Sayah, Catharina Prix, Elisabeth Wlasich, Olivia Wagemann, Sandra Loosli, Sonja Schönecker, Tobias Hoegen, Jolina Lombardi, Sarah Anderl-Straub, Adeline Rollin, Gregory Kuchcinski, Maxime Bertoux, Thibaud Lebouvier, Vincent Deramecourt, Beatriz Santiago, Diana Duro, Maria João Leitão, Maria Rosario Almeida, Miguel Tábuas-Pereira, Sónia Afonso

**Affiliations:** Dementia Research Centre, Department of Neurodegenerative Disease, UCL Queen Square Institute of Neurology, University College London, London, United Kingdom; Centre for Cognitive and Clinical Neuroscience, Division of Psychology, Department of Life Sciences, Medicine and Life Sciences, College of Health, Brunel University London, London, United Kingdom; Dementia Research Centre, Department of Neurodegenerative Disease, UCL Queen Square Institute of Neurology, University College London, London, United Kingdom; Dementia Research Centre, Department of Neurodegenerative Disease, UCL Queen Square Institute of Neurology, University College London, London, United Kingdom; Dementia Research Centre, Department of Neurodegenerative Disease, UCL Queen Square Institute of Neurology, University College London, London, United Kingdom; Centre for Medical Image Computing, Department of Medical Physics and Biomedical Engineering, University College London, London, United Kingdom; Department of Medical Statistics, London School of Hygiene and Tropical Medicine, London, United Kingdom; Dementia Research Centre, Department of Neurodegenerative Disease, UCL Queen Square Institute of Neurology, University College London, London, United Kingdom; Dementia Research Centre, Department of Neurodegenerative Disease, UCL Queen Square Institute of Neurology, University College London, London, United Kingdom; Dementia Research Centre, Department of Neurodegenerative Disease, UCL Queen Square Institute of Neurology, University College London, London, United Kingdom; Neuroradiological Academic Unit, Department of Brain Repair and Rehabilitation, UCL Queen Square Institute of Neurology, University College London, London, United Kingdom; Dementia Research Centre, Department of Neurodegenerative Disease, UCL Queen Square Institute of Neurology, University College London, London, United Kingdom; Centre for Medical Image Computing, Department of Medical Physics and Biomedical Engineering, University College London, London, United Kingdom; Martinos Center for Biomedical Imaging, Massachusetts General Hospital and Harvard Medical School, Charlestown, MA, USA; Computer Science and Artificial Intelligence Laboratory, Massachusetts Institute of Technology, Cambridge, MA, USA; Department of Neurology and Alzheimer center, Erasmus Medical Center Rotterdam, Rotterdam, The Netherlands; Department of Neurology and Alzheimer center, Erasmus Medical Center Rotterdam, Rotterdam, The Netherlands; Department of Neurology and Alzheimer center, Erasmus Medical Center Rotterdam, Rotterdam, The Netherlands; Centre for Neurodegenerative Disorders, Neurology Unit, Department of Clinical and Experimental Sciences, University of Brescia, Brescia, Italy; Department of Biomedical, Surgical and Dental Sciences, University of Milan, Milan, Italy; Fondazione IRCCS Ca’ Granda, Ospedale Maggiore Policlinico, Milan, Italy; Neurology Department, Hospital Clinic, Institut d’Investigacions Biomèdiques, Barcelona, Spain; Clinique Interdisciplinaire de Mémoire, Département des Sciences Neurologiques, CHU de Québec, Faculté de Médecine, Université Laval, Quebec City, QC, Canada; Hospital Universitario Donostia, San Sebastian, Spain; Division Translational Genomics of Neurodegenerative Diseases, Hertie Institute for Clinical Brain Research (HIH), University of Tübingen, Tübingen, Germany; German Center for Neurodegenerative Diseases (DZNE), Tübingen, Germany; Department NVS, Division of Neurogeriatrics, Karolinska Institutet, Stockholm, Sweden; Unit for Hereditary Dementia, Theme Aging, Karolinska University Hospital-Solna Stockholm, Stockholm, Sweden; Campbell Cognitive Neurology Research Unit, Sunnybrook Research Institute, Toronto, ON, Canada; Toronto Western Hospital, Tanz Centre for Research in Neurodegenerative Disease, Toronto, ON, Canada; Department of Clinical Neurosciences and Cambridge University Hospitals NHS Trust and Medical Research Council Cognition and brain Sciences Unit, University of Cambridge, Cambridge, United Kingdom; Laboratory for Cognitive Neurology, Department of Neurosciences, KU Leuven, Leuven, Belgium; Department of Clinical Neurological Sciences, University of Western Ontario, London, ON, Canada; Fondazione Istituto di Ricovero e Cura a Carattere Scientifico, Istituto Neurologico Carlo Besta, Milan, Italy; Faculty of Medicine, University of Lisbon, Lisbon, Portugal; Neurology Department, Centro Hospitalar e Universitário de Coimbra, Coimbra, Portugal; Department of Clinical Neurology, University of Oxford, Oxford, United Kingdom; Department of Neurology and Neurosurgery, McGill University, Montreal, QC, Canada; Division of Neuroscience and Experimental Psychology, Wolfson Molecular Imaging Centre, University of Manchester, Manchester, United Kingdom; Departments of Geriatric Medicine and Nuclear Medicine, University of Duisburg-Essen, Essen, Germany; Neurologische Klinik und Poliklinik, Ludwig-Maximilians-Universität, Germany; Neurologische Klinik und Poliklinik, Ludwig-Maximilians-Universität, Germany; German Center for Neurodegenerative Diseases (DZNE), Germany; Munich Cluster of Systems Neurology, Munich, Germany; Department of Neurology, University Hospital Ulm, Ulm, Germany; Department of Neuroscience, Psychology, Drug Research and Child Health, University of Florence, Florence, Italy; IRCCS Fondazione Don Carlo Gnocchi, Florence, Italy; Sorbonne Université, Paris Brain Institute—Institut du Cerveau– ICM, Inserm U1127, CNRS UMR 7225, AP-HP—Hôpital Pitié-Salpêtrière, Paris, France; Centre deréférence des démences rares ou précoces, IM2A, Département de Neurologie, AP-HP—Hôpital Pitié-Salpêtrière, Paris, France; Département de Neurologie, AP-HP—Hôpital Pitié-Salpêtrière, Paris, France; University Lille, Lille, France; Inserm 1172, Lille, France; CHU, CNR-MAJ, Labex Distalz, LiCENDLille, Lille, France; Dementia Research Centre, Department of Neurodegenerative Disease, UCL Queen Square Institute of Neurology, University College London, London, United Kingdom

**Keywords:** genetic frontotemporal dementia, MRI imaging, brain volumetry, diffusion imaging, presymptomatic stage

## Abstract

Biomarkers that can predict disease progression in individuals with genetic frontotemporal dementia are urgently needed. We aimed to identify whether baseline MRI-based grey and white matter abnormalities are associated with different clinical progression profiles in presymptomatic mutation carriers in the GENetic Frontotemporal dementia Initiative. Three hundred eighty-seven mutation carriers were included (160 *GRN*, 160 *C9orf72*, 67 *MAPT*), together with 240 non-carrier cognitively normal controls. Cortical and subcortical grey matter volumes were generated using automated parcellation methods on volumetric 3T T1-weighted MRI scans, while white matter characteristics were estimated using diffusion tensor imaging. Mutation carriers were divided into two disease stages based on their global CDR®+NACC-FTLD score: presymptomatic (0 or 0.5) and fully symptomatic (1 or greater). The *w*-scores in each grey matter volumes and white matter diffusion measures were computed to quantify the degree of abnormality compared to controls for each presymptomatic carrier, adjusting for their age, sex, total intracranial volume, and scanner type. Presymptomatic carriers were classified as ‘normal’ or ‘abnormal’ based on whether their grey matter volume and white matter diffusion measure *w*-scores were above or below the cut point corresponding to the 10th percentile of the controls. We then compared the change in disease severity between baseline and one year later in both the ‘normal’ and ‘abnormal’ groups within each genetic subtype, as measured by the CDR®+NACC-FTLD sum-of-boxes score and revised Cambridge Behavioural Inventory total score. Overall, presymptomatic carriers with normal regional *w*-scores at baseline did not progress clinically as much as those with abnormal regional *w*-scores. Having abnormal grey or white matter measures at baseline was associated with a statistically significant increase in the CDR®+NACC-FTLD of up to 4 points in *C9orf72* expansion carriers, and 5 points in the *GRN* group as well as a statistically significant increase in the revised Cambridge Behavioural Inventory of up to 11 points in *MAPT*, 10 points in *GRN,* and 8 points in *C9orf72* mutation carriers. Baseline regional brain abnormalities on MRI in presymptomatic mutation carriers are associated with different profiles of clinical progression over time. These results may be helpful to inform stratification of participants in future trials.

## Teaser

Bocchetta *et al*. quantified brain anomalies on MRI in a large cohort of *C9orf72*, *MAPT*, and *GRN* mutation carriers. They defined the imaging markers associated with the largest clinical and behavioral changes over one year in presymptomatic carriers, providing important data to inform participants’ stratification in trials.

## Graphical Abstract

**Graphical abstract:**
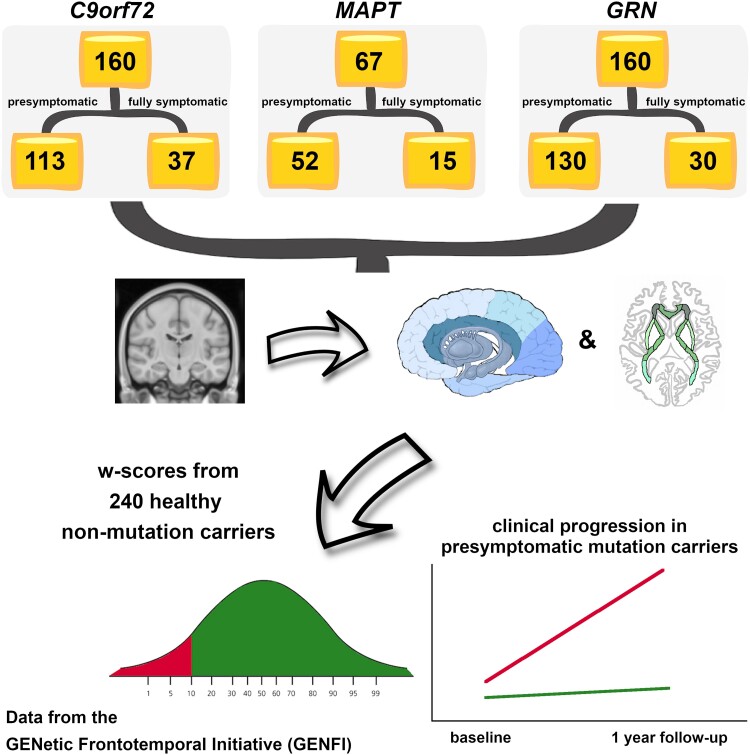

## Introduction

Genetic frontotemporal dementia (FTD) is a progressive and heterogeneous neurodegenerative disease most frequently caused by an autosomal dominant genetic mutation in the microtubule-associated protein tau (*MAPT*), progranulin (*GRN*), or chromosome 9 open reading frame 72 (*C9orf72*).^1^ Changes in grey and white matter regions measured by magnetic resonance imaging (MRI) have been reported many years before symptoms develop in previous studies,^2-4^ but the exact relationship of such brain changes to clinical progression is yet to be fully understood. This is particularly relevant in current research, considering the need for robust biomarkers to allow accurate measurement of disease onset and progression in the context of clinical trials.

Using data from the GENetic FTD Initiative (GENFI) cohort, we aimed to localize and quantify the specific pattern of subregional grey and white matter abnormalities in the prodromal and symptomatic stages of genetic FTD, and how these abnormalities relate to progression of symptoms in presymptomatic mutation carriers.

## Materials and methods

At the time of the fifth data freeze in the GENFI study, 850 participants had been recruited as part of the second phase (03 March 2015–31 May 2019) across 24 centers in the United Kingdom, Canada, Italy, The Netherlands, Sweden, Portugal, Germany, France, Spain, and Belgium, of whom 710 had volumetric T1- and diffusion-weighted MRI acquired on a 3T scanner. Eighty three of these participants were excluded as their scans were of unsuitable quality due to motion, incomplete spatial coverage or other imaging artifacts, for pathology unlikely to be attributed to FTD, or if they were carriers of mutations in one of the rarer genetic causes of FTD. All the remaining 627 participants were known to be either a carrier of a pathogenic expansion in *C9orf72* or of a pathogenic mutation in *GRN* or *MAPT* (*n* = 387), or were non-carrier first-degree relatives (*n* = 240), who therefore acted as controls within the study. Participants have been screened and genotyped at their local sites for the most common pathogenic genetic mutations for FTD. All aspects of the study were approved by the local ethics committee for each of the GENFI sites, and written informed consent was obtained from all participants.

All participants underwent a standardized clinical assessment as described previously.^2^ This included the CDR® plus NACC FTLD,^5^ a measure of disease severity, from which both a global score and a sum of boxes score can be calculated. The global score can be used to stage mutation carriers, with those with a score of 0 or 0.5 considered as ‘presymptomatic’, and those with a score of 1, 2, or 3 considered as ‘fully symptomatic’ (Table 1). In addition, the revised version of the Cambridge Behavioural Inventory (CBI-R)^6^ was also completed as a measure of behavioral impairment.

**Table 1:** Demographic and clinical characteristic of the cohort divided by genetic group and CDR®+NACC FTLD global scores

		*C9orf72* expansion carriers		*MAPT* mutation carriers		*GRN* mutation carriers	
CDR®+NACC FTLD global score	Non-carriers	≤0.5	≥1	≤0.5	≥1	≤0.5	≥1
*N*	240	113	47	52	15	130	30
Age, year (mean and SD)	44.8(12.2)	45.0(11.5)	63.5(7.4)	41.1(10.6)	59.2(9.3)	46.5(12.2)	63.3(8.1)
Sex, male (%)	103(42.9%)	48(42.5%)	31(66.0%)	21(40.4%)	9(60.0%)	48(36.9%)	14(46.7%)
Clinical phenotype	N/A	N/A	36 bvFTD, 4 FTD-ALS, 2 ALS, 2 PPA, 1 PSP, 1 Dementia-NOS, 1 Other	N/A	13 bvFTD, 1 PPA, 1 Dementia-NOS	N/A	16 bvFTD, 12 PPA, 1 CBS, 1 Other
CDR®+NACC FTLD sum of boxes score (mean and SD) (baseline/follow-up)	N/A	0.2(0.5)/0.9(2.5)	N/I	0.3(0.7)/0.6(1.3)	N/I	0.2(0.5)/0.8(2.8)	N/I
CBI-R total score (mean and SD) (baseline/follow-up)	N/A	9.0(9.5)/9.4(14.5)	N/I	6.8(7.8)/9.0(13.4)	N/I	5.2(8.5)/7.0(13.5)	N/I

Abbreviations: SD, standard deviation; N/A, not applicable; N/I, not included in the analyses; FTD, frontotemporal dementia; bvFTD, behavioural variant FTD; PPA, primary progressive aphasia; NOS, not otherwise specified; CBS, corticobasal syndrome; PSP, progressive supranuclear palsy; ALS, amyotrophic lateral sclerosis; CBI-R, revised version of the Cambridge Behavioural Inventory.

Participants underwent MRI scans on five types of 3T system from different vendors (Siemens Trio, Siemens Skyra, Siemens Prisma, Philips Achieva, GE Discovery MR750). Specific acquisition parameters are reported in the Supplementary Table 1.

### T1-weighted processing

**Figure 1:**
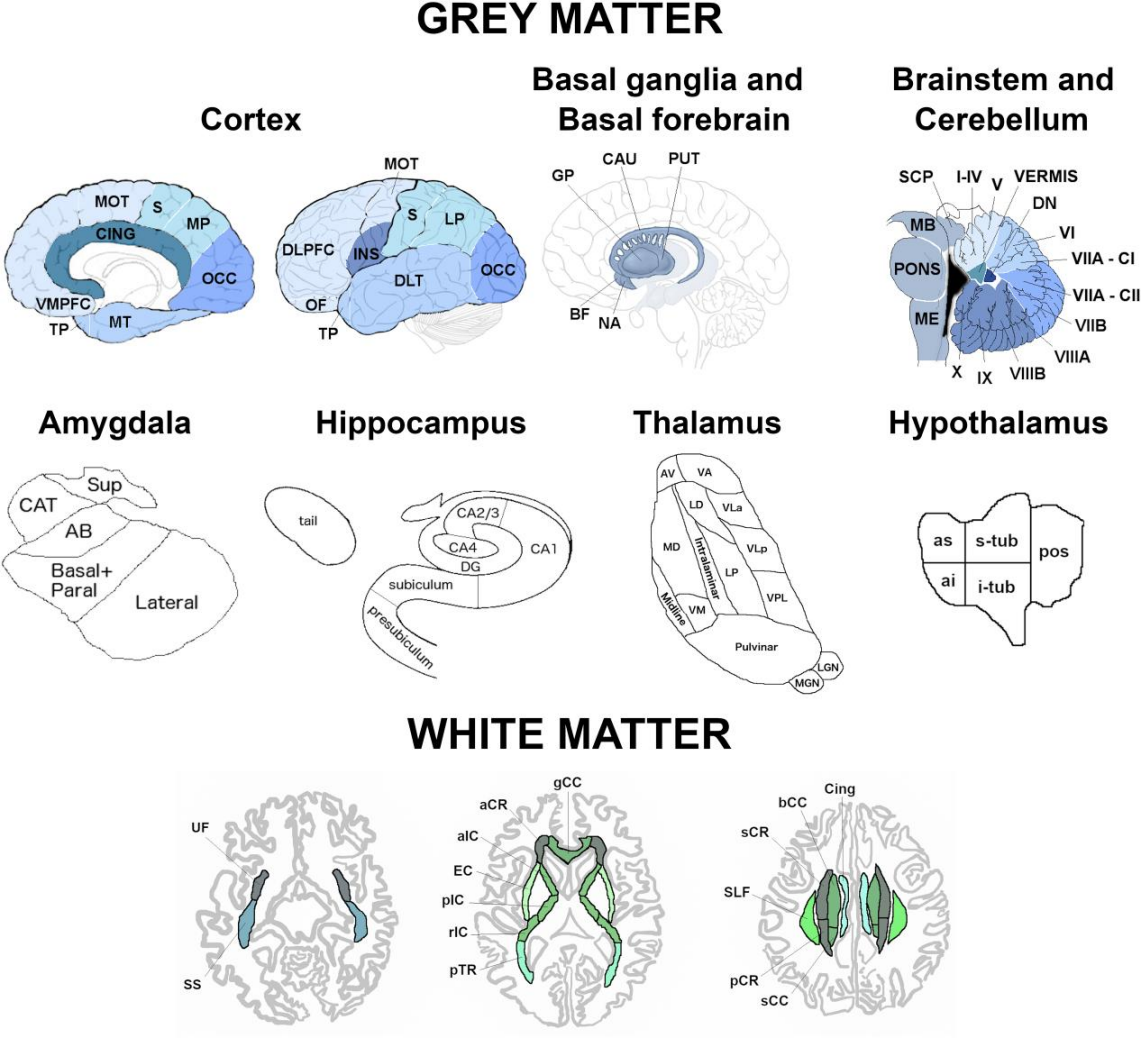
**Regions of interest used in the grey and white matter analyses.** Abbreviations. *Cortex*: VMPFC, ventromedial prefrontal; TP, temporal pole; MT, medial temporal; CING, cingulate; MOT, motor; S, sensory; MP, medial parietal; OCC, occipital; DLPFC, dorsolateral prefrontal; OF, orbitofrontal; INS, insula; DLT, dorsolateral temporal; LP, lateral parietal. *Basal ganglia and basal forebrain*: GP, pallidum; CAU, caudate; PUT, putamen; BF, basal forebrain; NA, nucleus accumbens. *Brainstem and cerebellum*: SCP, superior cerebellar peduncle; MB, midbrain; ME, medulla; VIIA-CI lobule, VIIA-Crus I; VIIA-CII lobule, VIIA-Crus II; DN, deep nuclei. *Amygdala*: CAT, cortico-amygdaloid transition area; Sup, superficial nuclei; AB, accessory basal nucleus. *Hippocampus*: DG, dentate gyrus; CA, cornu ammonis. *Thalamus*: AV, anteroventral; VA, ventral anterior; LD, laterodorsal; VLa, ventral lateral anterior; MD, mediodorsal; LP, lateral posterior; VLp, ventral lateral posterior; VPL, ventral posterolateral; VM, ventromedial; LGN, lateral geniculate nucleus; MGN, medial geniculate nucleus. *Hypothalamus*: as, anterior superior; ai, anterior inferior; s-tub, superior tuberal; i-tub, inferior tuberal; pos, posterior. *White matter tracts*: UF, uncinate fasciculus; SLF, superior longitudinal fasciculus; Cing, cingulum; SS, sagittal stratum; pTR, posterior thalamic radiation; aCR, anterior corona radiata; pCR, posterior corona radiata; sCR, superior corona radiata; EC, external capsule; aIC, anterior part of the internal capsule; pIC, posterior part of the internal capsule; rIC, retrolenticular part of the internal capsule; gCC, genu of the corpus callosum; bCC, body of the corpus callosum; sCC, splenium of the corpus callosum.

The processing was performed as previously described.^7^ In brief, volumetric MRI scans were first bias field corrected and whole brain parcellated using the geodesic information flow (GIF) algorithm,^8^ which is based on atlas propagation and label fusion. We combined regions of interest (ROIs) to calculate grey matter (GM) volumes of 13 ROIs of the cortex (Fig. 1): orbitofrontal, dorsolateral (DLPFC) and ventromedial prefrontal (VMPFC), motor, insula, temporal pole, dorsolateral and medial temporal, cingulate, sensory, medial and lateral parietal, and occipital cortex. We used GIF and customized versions of specific Freesurfer modules^9-12^ that accept the GIF parcellation as inputs^13-16^ to calculate individual volumes for the following subcortical ROIs (Fig. 1): (i) basal ganglia (nucleus accumbens, caudate, putamen, and globus pallidus, (ii) basal forebrain, (iii) amygdala (5 regions: lateral nucleus, basal and paralaminar nucleus, accessory basal nucleus, cortico-amygdaloid transition area and the superficial nuclei), (iv) hippocampus (7 regions: cornu ammonis CA1, CA2/CA3, CA4, dentate gyrus, subiculum, presubiculum, tail), (v) thalamus [14 regions: anteroventral, laterodorsal (LD), lateral posterior, ventral anterior, ventral lateral anterior, ventral lateral posterior, ventral posterolateral, ventromedial, intralaminar, midline, mediodorsal, lateral geniculate nucleus, medial geniculate (MGN) and pulvinar]. We computed the volumes for the hypothalamus [5 regions: anterior superior, anterior inferior, superior tuberal (s-tub), inferior tuberal (i-tub), posterior] using the deep convolutional neural network method described in Billot *et al*.^17^ We also parcellated the cerebellum (separated into 12 regions: lobules I-IV, V, VI, VIIa-Crus I, VIIa-Crus II, VIIb, VIIIa, VIIIb, IX, X, vermis, and deep nuclei),^18,19^ and brainstem (superior cerebellar peduncle, medulla, pons, and midbrain).^10^ We calculated the whole brain volume by summing the white matter (WM) and GM regions extracted from GIF. We summed left and right volumes, and we computed the total intracranial volume (TIV) with SPM12 v6470 (Statistical Parametric Mapping, Wellcome Trust Centre for Neuroimaging, London, UK) running under Matlab R2014b (Math Works, Natick, MA, USA).^20^ All segmentations were visually checked for quality with only one subject excluded from the cerebellar analyses due to the presence of an arachnoid cyst.

### DTI acquisition and processing

The preprocessing was carried out with a combination of source tools described below, wrapped up by NiftyPipe (http://cmictig.cs.ucl.ac.uk/wiki/index.php/NiftyPipe) software packages. First, the multiple diffusion-weighted image (DWI) acquisitions were merged with the FMRIB Software Library (FSL, v5.0.4).^21^ Then, the images were corrected for eddy-current distortion and motion by performing an affine co-registration between the DWIs and the averaged b0 images, using FSL eddy function.^22^ Susceptibility-induced image distortions were subsequently corrected using a unified field map and image-registration-based approach.^23^ We used the subject-specific structural T1-weighted image as the reference space, and the TIV binary mask extracted from GIF to restrict the analyses to the brain, and to improve the registration. Niftyfit^24^ was used for the diffusion tensor fitting, which was estimated using the weighted least square method. Before the groupwise registration, the tensors were visually checked and prepared according to the approach reported in (http://dti-tk.sourceforge.net/pmwiki/pmwiki.php? n=Documentation.BeforeReg). DTI-TK (https://dti-tk.sourceforge.net/pmwiki/pmwiki.php)^25-28^ was used to spatially normalize the diffusion tensor volumes to a population-specific tensor template,^27,29^ where the ‘IXI aging template’^30-32^ was used for the template initialization, from an initial rigid registration, followed by non-linear registration (http://dti-tk.sourceforge.net/pmwiki/pmwiki.php?n=Documentation.Registration).^27,32^

We created maps of fractional anisotropy (FA) and mean diffusivity (MD) for each diffusion tensor image in the groupwise space. Using NiftyReg,^33^ we registered the FA image from the study-specific template with the John Hopkins University (JHU)^34^ FA image provided in FSL, applying an affine transformation first, followed by a non-linear registration using a B-spline. The mean FA and MD were then extracted for the following WM tracts from the JHU atlas^34^ using DTI-TK (Fig. 1): uncinate fasciculus (UF), superior longitudinal fasciculus (SLF), cingulum, sagittal stratum (SS), posterior thalamic radiation (pTR), anterior (aCR), posterior (pCR) and superior (sCR) corona radiata, external capsule (EC), anterior (aIC), posterior (pIC) and retrolenticular part (rIC) of the internal capsule, and genu (gCC), body (bCC) and splenium of the corpus callosum (sCC). Left and right values were averaged to obtain one bilateral value per metric (FA and MD) per tract.

### Statistical analyses

We computed *w*-scores for each of the volumes and diffusion indexes for the GM and WM ROIs. The *w*-score is a metric that quantifies the extent of abnormalities in each index for each mutation carrier after adjusting for the effects of age, sex, TIV, and scanner type. To calculate the *w*-score, first linear regression models were carried out in controls to relate the value of each index to age, sex, TIV, and scanner type. After fitting the model, predicted values of the index were produced for the mutation carriers using the control model equation, given the mutation carriers age, sex, TIV, and the scanner type. Finally, the *w*-scores were calculated using the following formula:


$${\mathrm{wscore}}_{i}=\frac{x_{i}-{\hat{x}}_{l}}{\sigma }$$

where wscore_*i*_ is the *w*-score for the *i*th mutation carrier, $x_{i}$ is the observed value of the index for the *i*th mutation carrier, ${\hat{x}}_{i}$ is the predicted value of the index for the *i*th mutation carrier based on the control linear regression model, given the mutation carriers age, sex, TIV, and the scanner type, and σ is the square root of the residual variance from the linear regression model in controls. The *w*-scores have a similar interpretation to *Z*-scores: in the control group, they have a mean value of 0 and a standard deviation of 1; a *w*-score of -1.960 corresponds to the 2.5th percentile of the controls, -1.645 to the 5th percentile, -1.282 to the 10th percentile, and -0.675 to the 25th percentile.

Statistical analyses were performed in Stata v.14 (Stata Statistical Software: College Station, TX: StataCorp LP) and SPSS v.26 software (SPSS Inc., Chicago, IL, USA). First, we calculated non-parametric percentile 95% confidence intervals using bootstrapping with 1000 replicates (as not all variables were normally distributed) to verify whether the GM and WM *w*-scores in the presymptomatic and symptomatic (as defined by CDR® plus NACC FTLD) subgroups within each genetic groups (*C9orf72*, *MAPT*, *GRN*) were each significantly different from 0, indicating the mean in the genetic group was below the mean of controls (or above 0 in the case of MD).

Then, we focused on the presymptomatic carriers (excluding those scoring CDR® plus NACC FTLD≥1) and for each of the cortical, whole subcortical and WM ROIs we separated those with a *w*-score below and above -1.282 (or for MD above and below 1.282) (‘abnormal’ versus ‘normal’, with ‘abnormal’ corresponding to the 10th percentile of the controls), and we compared their CDR® plus NACC FTLD sum of boxes scores and their CBI-R total scores after one year. To investigate whether there was a difference in the clinical and behavioral scores over time in the ‘abnormal’ versus ‘normal’ groups for each of the GM and WM ROIs, we performed a Wilcoxon Signed Rank exact test to compare baseline to follow-up scores for the presymptomatic carriers who had follow-up visits available at 12 months (*C9orf72 n* = 56; *MAPT n* = 32; *GRN n* = 69). We excluded groups with <3 carriers and we considered with caution non-significant results on groups with <6 carriers, as it is not possible for these comparisons to reach statistical significance on the Wilcoxon Signed Rank test.

### Data Availability

Data will be shared according to the GENFI data sharing agreement, after review by the GENFI data access committee with final approval granted by the GENFI steering committee.

## Results

### Baseline GM volumes

The results on the analyses for the *w*-scores on GM volumes at baseline are described in detail in the Supplementary material and in Supplementary Table 2.

**Figure 2:**
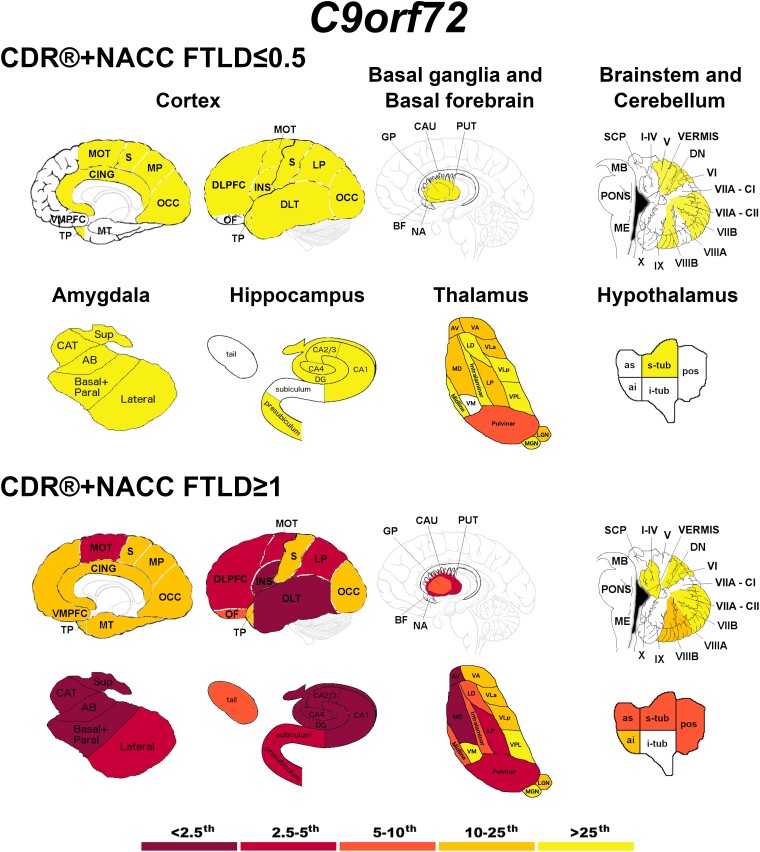
**Pattern of grey matter involvement in *C9orf72* for the stages defined by CDR®+NACC FTLD global scores.** The color map indicates the percentile corresponding to the mean *w*-scores in each group (*C9orf72* ≤ 0.5/≥1: n = 113/47), when these were statistically abnormal (i.e. significantly different from 0, *t*-test) when compared to controls (*n* = 240).

Briefly*, C9orf72* expansion carriers showed the most widespread GM differences (Fig. 2), even at a presymptomatic stage. In particular, the thalamus was the structure with the most abnormal regions in presymptomatically, with the pulvinar showing *w*-scores below the 10th percentile of the controls.

**Figure 3:**
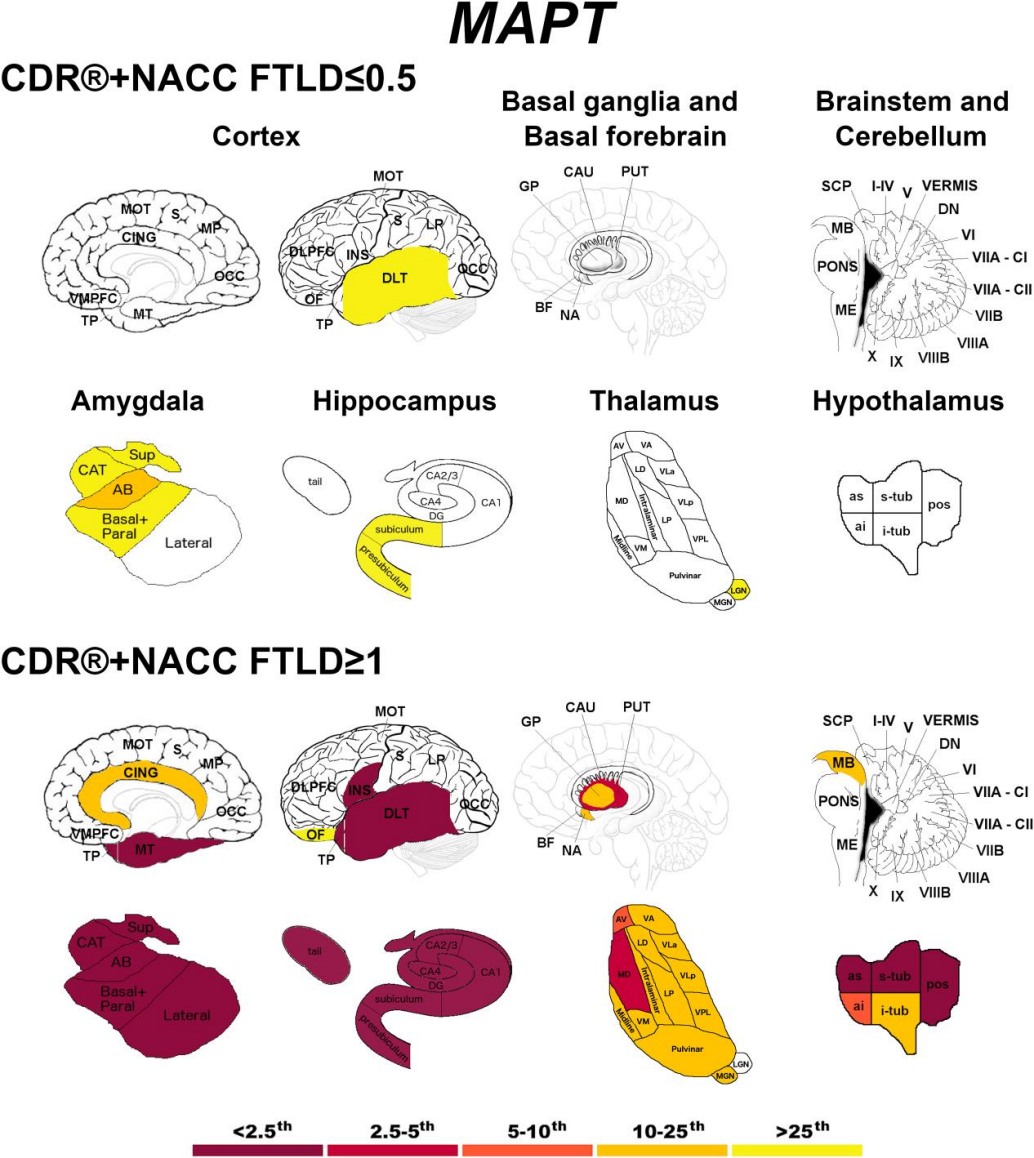
**Pattern of grey matter involvement in *MAPT* for the stages defined by CDR®+NACC FTLD global scores.** The color map indicates the percentile corresponding to the mean *w*-scores in each group (*MAPT* ≤0.5/≥1: *n* = 52/15), when these were statistically abnormal (i.e. significantly different from 0, *t*-test) when compared to controls (*n* = 240).

Presymptomatic *MAPT* mutation carriers showed localized abnormal *w*-scores in the dorsolateral temporal cortex and in regions of the amygdala, hippocampus and thalamus (Fig. 3). At the fully symptomatic stage, values were extremely low (<2.5th percentile) in all the temporal cortex, amygdala, hippocampus and insula, and in some regions of the hypothalamus (Fig. 3).

**Figure 4:**
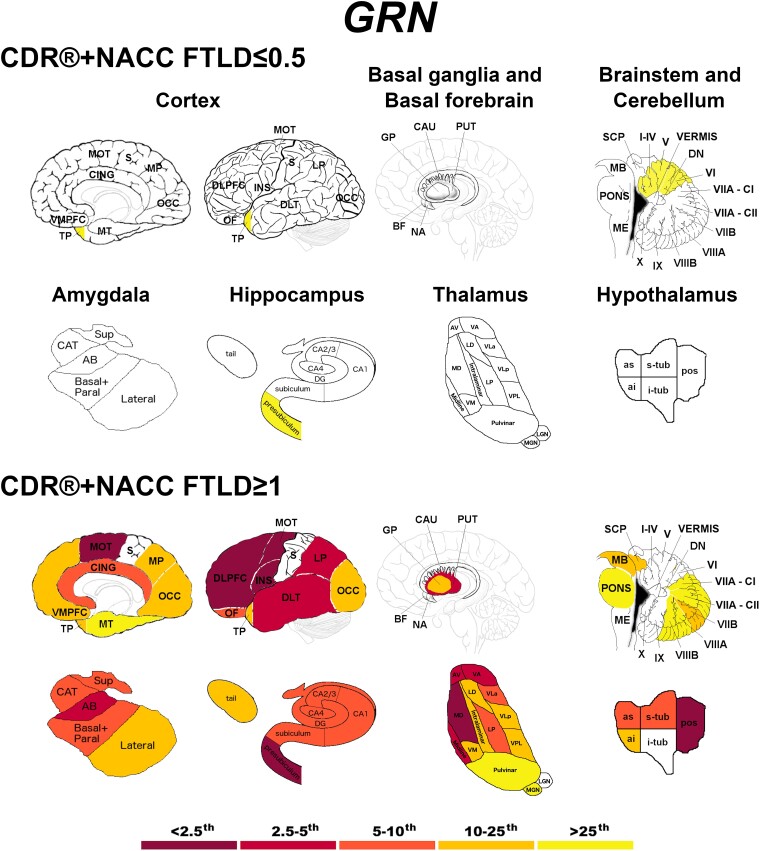
**Pattern of grey matter involvement in *GRN* for the stages defined by CDR®+NACC FTLD global scores.** The color map indicates the percentile corresponding to the mean *w*-scores in each group (*GRN* ≤0.5/≥1: *n* = 130/30), when these were statistically abnormal (i.e. significantly different from 0, *t*-test) when compared to controls (*n* = 240).

Presymptomatic *GRN* mutation carriers showed significantly lower values in the temporal pole, the presubiculum, and the anterior superior cerebellum (Fig. 4). Fully symptomatic carriers showed widespread cortical and subcortical involvement, with extremely low *w*-scores (<2.5th percentile) in the DLPFC, insula and motor cortex, and in the presubiculum, mediodorsal thalamus and posterior hypothalamus (Fig. 4).

### Baseline diffusion WM indices

**Figure 5:**
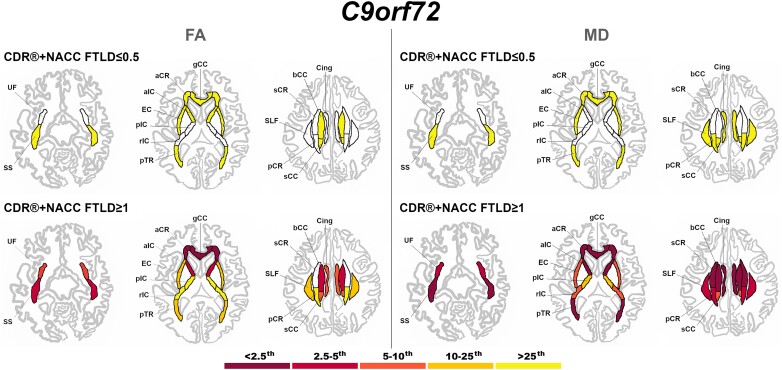
**Pattern of white matter involvement in *C9orf72* for the stages defined by CDR®+NACC FTLD global scores.** FA indexes are reported on the left side of the figure, while MD on the right. The color map indicates the percentile corresponding to the mean *w*-scores in each group (*C9orf72* ≤ 0.5/≥1: *n* = 113/47), when these were statistically abnormal (i.e. significantly different from 0, *t*-test) when compared to controls (*n* = 240).

Presymptomatic *C9orf72* expansion carriers showed both FA and MD values significantly different than controls in the SS, corpus callosum (genu and body), pTR, aCR, and EC (Fig. 5). Symptomatic *C9orf72* expansion carriers showed extremely abnormal values (<2.5th percentile) in the gCC and aCR for FA, and in the SS, corpus callosum (genu and body), aCR, sCR, cingulum, pTR, and aIC for MD (Fig. 5).

**Figure 6:**
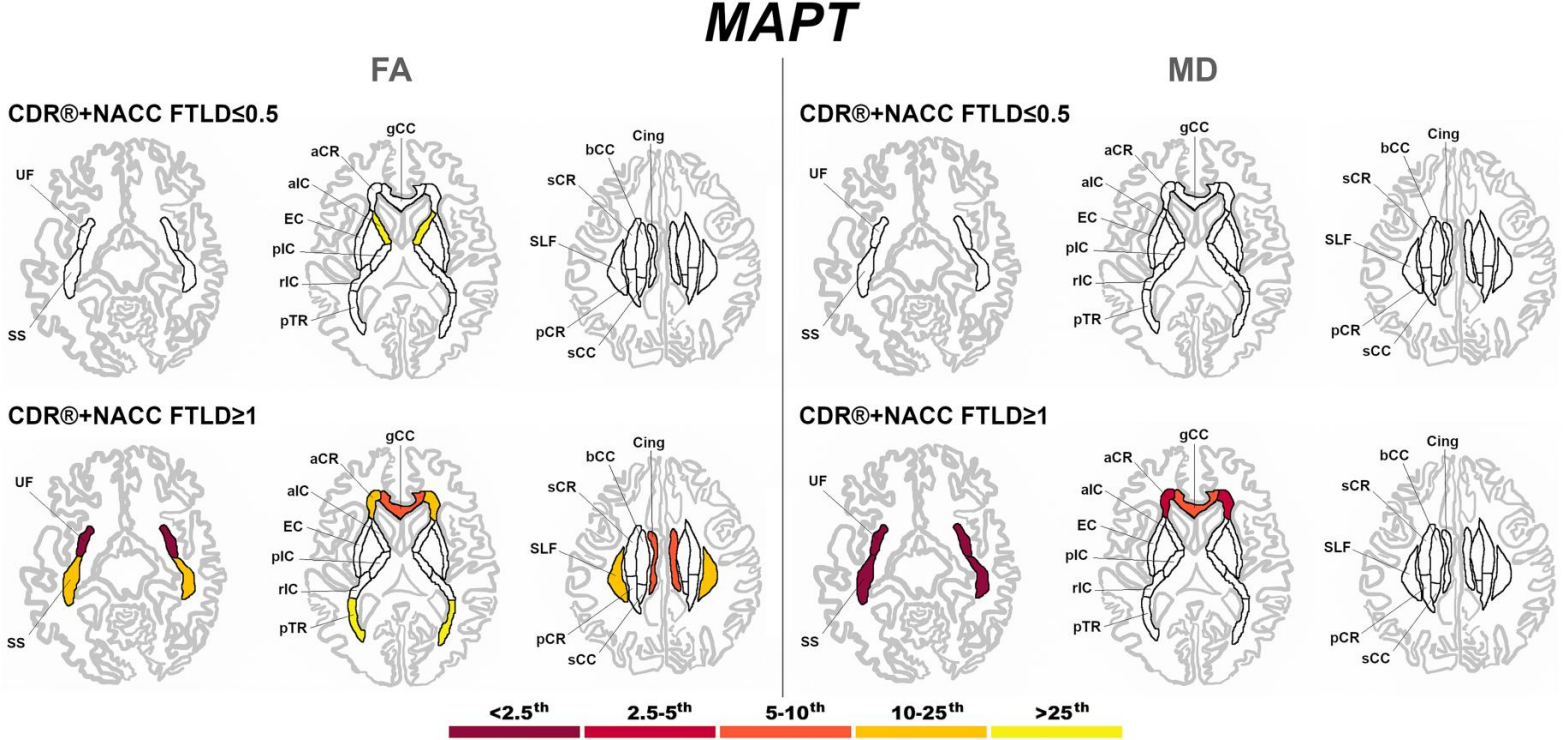
**Pattern of white matter involvement in *MAPT* for the stages defined by CDR®+NACC FTLD global scores.** FA indexes are reported on the left side of the figure, while MD on the right. The color map indicates the percentile corresponding to the mean *w*-scores in each group (*MAPT* ≤0.5/≥1: *n* = 52/15), when these were statistically abnormal (i.e. significantly different from 0, *t*-test) when compared to controls (*n* = 240).

Presymptomatic *MAPT* mutation carriers only showed significantly lower FA than controls in the aIC (Fig. 6). Once symptoms were present, *MAPT* mutation carriers showed extremely abnormal values (<2.5th percentile) for the UF (both FA and MD), and SS (MD only) (Fig. 6).

**Figure 7:**
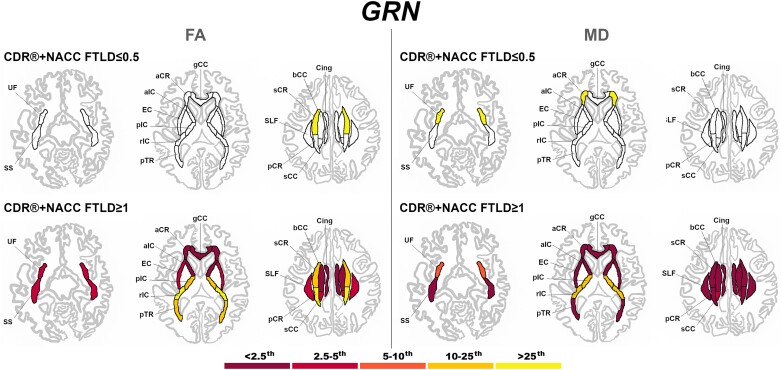
**Pattern of white matter involvement in *GRN* for the stages defined by CDR®+NACC FTLD global scores.** FA indexes are reported on the left side of the figure, while MD on the right. The color map indicates the percentile corresponding to the mean *w*-scores in each group (*GRN* ≤0.5/≥1: *n* = 130/30), when these were statistically abnormal (i.e. significantly different from 0, *t*-test) when compared to controls (*n* = 240).

At a presymptomatic stage, *GRN* mutation carriers showed significantly lower FA than controls in the sCR, and significantly higher MD than controls in the UF and aCR (Fig. 7). Fully symptomatic *GRN* mutation carriers showed abnormal FA and MD values in all tracts, with extremely abnormal values (<2.5th percentile) in the corpus callosum (genu and body), cingulum, aIC, and aCR for FA, and in nearly all tracts for MD (Fig. 7).

Detailed description of the results is reported in the Supplementary material and in Supplementary Table 3.

### Progression

**Figure 8:**
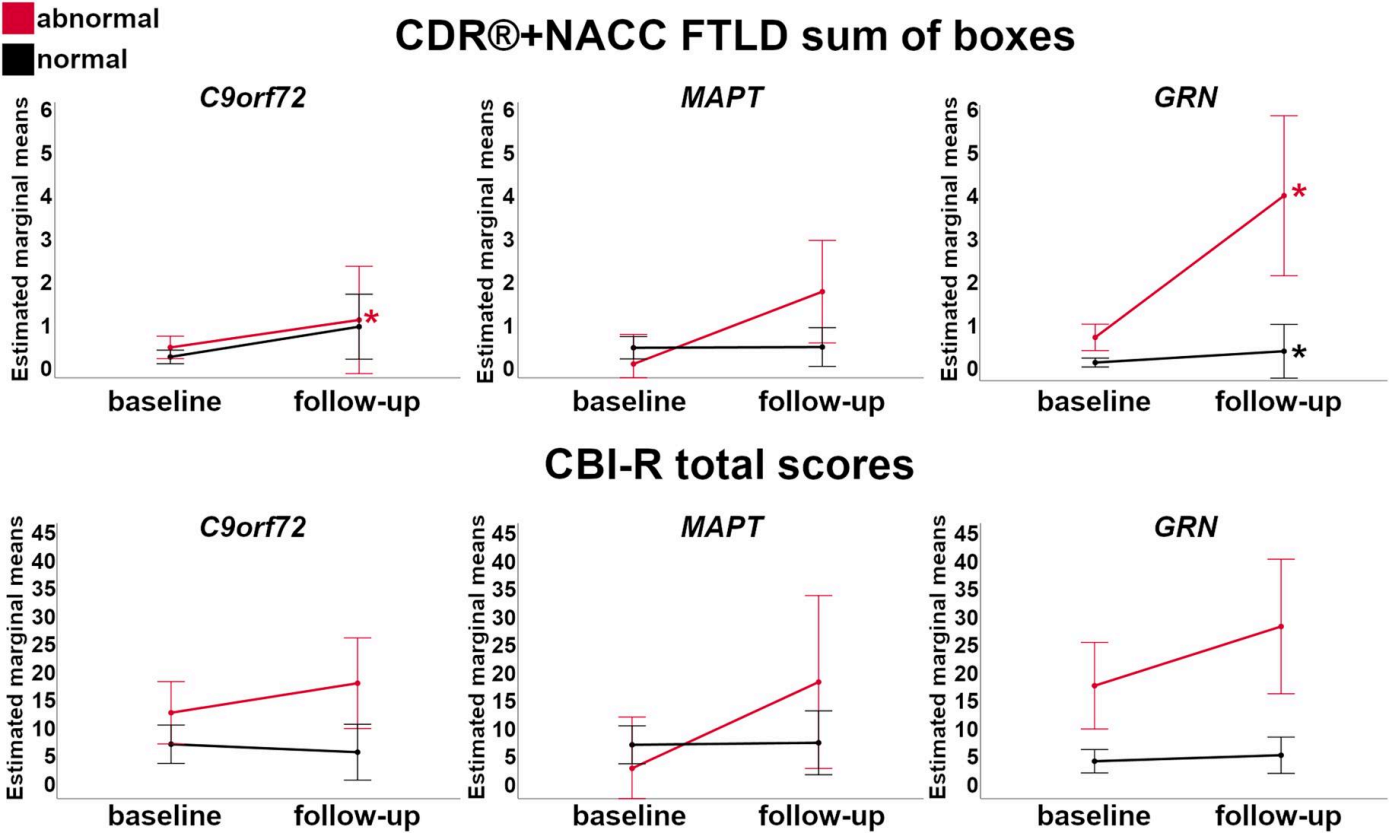
**Longitudinal changes in the CDR®+NACC FTLD sum of boxes scores (first row) and CBI-R total scores (second row) in the presymptomatic mutation carriers for those with *w*-scores of the whole brain volume above (‘normal’ in black) and below (‘abnormal’ in red) the 10th percentile of the controls.** Asterisks indicate a significant difference in progression between visits within the two groups at Wilcoxon Signed Rank exact test (*P* ≤ 0.05). Bars indicate the 95% confidence intervals of the mean. Analyses were performed on: *C9orf72 n* = 56; *MAPT n* = 32; *GRN n* = 69.

Figure 8 shows the longitudinal changes in the CDR®+NACC FTLD sum of boxes and CBI-R total scores over one year in the presymptomatic mutation carriers with *w*-scores of the *whole brain volume* above (‘normal’) and below (‘abnormal’) the 10th percentile of the controls. For the CDR®+NACC FTLD sum of boxes, *C9orf72* and *GRN* mutation carriers showed significant increases within the ‘abnormal brain’ subgroups (1 and 3 points respectively), with *GRN* mutation carriers also showing significant increases of 0.3 points in the subgroup with ‘normal’ brain at baseline. The increase in 2 points for *MAPT* mutation carriers did not reach statistical significance, as there were only 4 carriers in the ‘abnormal brain’ subgroup. Although both the *MAPT* and *GRN* ‘abnormal brain’ subgroups showed a substantial increase in CBI-R of 16 and 11 points, respectively, this was not statistically significant due to the small sample sizes for analysis of this measure (*n* = 3 and 4). Overall, the magnitude of clinical changes in *C9orf72* expansion carriers was smaller than what seen in *MAPT* and *GRN* mutation carriers.

Supplementary Tables 4 and 5 show the results of the longitudinal change in both the CDR®+NACC FTLD sum of boxes and CBI-R total scores across the three genetic groups for all the individual GM and WM ROIs. Below, we discuss the ROIs which showed the largest significant change in scores (i.e. most clinical progression) over time within the three genetic groups.

#### *C9orf72* expansion carriers

**Figure 9:**
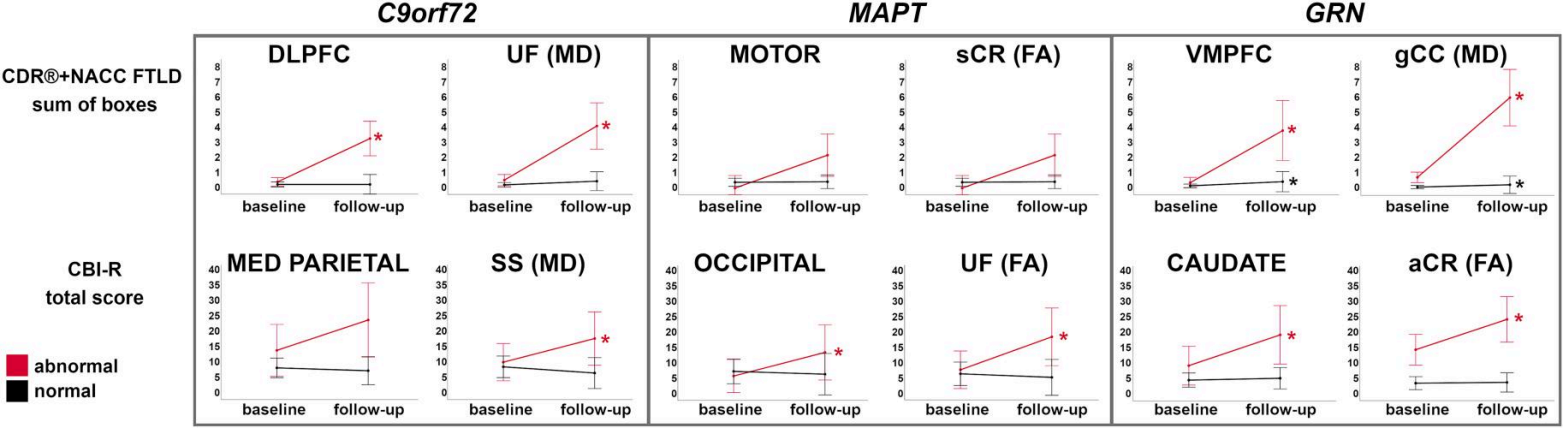
**Largest longitudinal changes in the CDR®+NACC FTLD sum of boxes scores (first row) and CBI-R total scores (second row) in the presymptomatic mutation carriers for those with ‘normal’ and ‘abnormal’ *w*-scores for GM and WM regions.** *Y*-axis represents estimated marginalized means. Asterisks indicate a significant difference in progression between visits within the two groups at Wilcoxon Signed Rank exact test (*P* ≤ 0.05). Bars indicate the 95% confidence intervals of the mean. Analyses were performed on: *C9orf72 n* = 56; *MAPT n* = 32; *GRN n* = 69. Abbreviations. DLPFC, dorsolateral prefrontal; VMPFC, ventromedial prefrontal; MED PARIETAL, medial parietal, FA, fractional anisotropy; MD, mean diffusivity; UF, uncinate fasciculus; SS, sagittal stratum; gCC, genu of the corpus callosum; sCR, superior corona radiata; aCR, anterior corona radiata.

The ROIs in which *w*-score abnormalities at baseline resulted in the highest significant increase in the CDR®+NACC FTLD sum of boxes score were the DLFPC and motor cortex among the GM ROIs (+3 points, *P*-value ≤ 0.016, Supplementary Tables 4 and 5), and the MD in the UF (+4, *P*-value = 0.031), gCC and aCR (+3, *P*-value ≤ 0.031), together with the FA in the UF (+3, *P*-value = 0.031) among the WM diffusion indexes (Supplementary Tables 4 and 5, Fig. 9). Except for a few regions (Supplementary Tables 4 and 5), the subgroups with ‘normal’ GM and WM ROIs at baseline showed a statistically significant increase no greater than 1 point.

The *w*-scores that led to the largest change over time in the CBI-R total score were the MD in the SS and the FA in the cingulum (6–8 points, *P*-value ≤ 0.047), with similar values for the MD in the EC and gCC, although not reaching statistical significance as only five presymptomatic carriers were available for this analysis (Fig. 9 and Supplementary Tables 4 and 5). For none of the GM ROIs, *w*-score abnormalities at baseline resulted in a statistically significant increase. However, the medial parietal, cingulate, and nucleus accumbens led to a large change over time (7–10 points), which did not reach statistical significance given the small sample of carriers (*n* = 3 and 5).

Carriers with normal regional *w*-scores at baseline did not progress on the CBI-R total scores after 12 months.

#### *MAPT* mutation carriers

No statistically significant increase was found for the CDR®+NACC FTLD total score, which may be largely due to the small numbers in the abnormal subgroups. However, when looking at which abnormal *w*-scores resulted in the highest increase of 2 points, these were the motor, putamen, and VMPFC, and the following among the WM diffusion indexes: gCC (both FA and MD), rIC, sCR, pCR, pTR, EC, SS, SLF (FA), and bCC and aCR (MD) (Fig. 9 and Supplementary Tables 4 and 5).

Abnormal *w*-scores values in the occipital cortex and in the FA in the UF led to a significant increase of respectively 8 and 11 points in the CBI-R total scores over a year (*P*-value ≤ 0.035, Fig. 9 and Supplementary Tables 4 and 5). Other large increases, despite not reaching statistically significance, were seen in the abnormal values for the hypothalamus (+19), VMPFC (+17), putamen and motor cortex (+16), and for the FA in the gCC, pTR, SS (+15) and SLF (+13) (Fig. 9 and Supplementary Tables 4 and 5).

Carriers with normal regional *w*-scores at baseline did not progress on the CDR®+NACC FTLD or CBI-R total scores after 12 months.

#### *GRN* mutation carriers

The abnormal ROIs which showed the largest statistically significant increase in CDR®+NACC FTLD sum of boxes scores were the MD in the gCC and EC (+4–5, *P*-value ≤ 0.031), followed by MD in the aCR, FA in the aIC and aCR, and VMPFC, motor, lateral parietal, cingulate and hypothalamus (+3, *P*-value ≤ 0.031) (Fig. 9 and Supplementary Tables 4 and 5). Moreover, the small sample of carriers with abnormal *w*-scores in the DLPFC and globus pallidus led to a large change over time (+5–6 points).

The subgroups with ‘normal’ GM and WM ROIs at baseline (except for MD in the sCC and aCR) showed statistically significant increase no greater than 1 point (Fig. 9 and Supplementary Tables 4 and 5).

For the CBI-R total scores, a statistically significant increase of 10 points was seen if the baseline *w*-scores for the caudate or the FA in aCR were abnormal (*P*-value ≤ 0.046), while a statistically significant increase of 8 and 9 points if the SLF (MD) and hypothalamus *w*-scores were abnormal (*P*-value ≤ 0.016) (Fig. 9 and Supplementary Tables 4 and 5). The small sample of carriers with abnormal *w*-scores at baseline also led to a large change over time, specifically in the globus pallidus (+18 points), hippocampus and gCC (MD) (+12), and DLPFC (+11).

The subgroups with ‘normal’ ROIs at baseline in the following regions were showing a statistically significant increase of 1 or 2 points: VMPFC, dorsolateral temporal, temporal pole, medial parietal, insula, cerebellum, basal forebrain; bCC, sCC, pCR, cingulum (FA); and aIC, pIC, pTR, SS (MD).

## Discussion

Using *in vivo* volumetric and diffusion MR imaging, we have quantified and localized the pattern of brain anomalies in a large cohort of presymptomatic and symptomatic carriers of *C9orf72*, *MAPT*, and *GRN* mutations. Moreover, we were able to define which neuroimaging markers were associated with the largest clinical and behavioral changes over one year in presymptomatic mutation carriers.

*C9orf72* expansion carriers showed the earliest and most widespread abnormalities in the brain, with the pulvinar and its posterior WM tracts being the most affected regions at the presymptomatic stage. These findings confirm what has been reported by previous studies, and in line with the role that the pulvinar plays in the development of psychotic symptoms in *C9orf72*.^3,7,35-41^ The presence of such early and widespread changes in *C9orf72* could be linked to an abnormal development in the brain networks, or to a very early neurodegenerative process, as suggested by Lee *et al*.^35^

*MAPT* mutation carriers were confirmed to have early and very localized abnormality in the mediotemporal lobe, especially in the medial amygdala, and in regions linked to the limbic network.^7,42^ The WM tracts mainly affected in *MAPT* mutation carriers are the UF, cingulum, SS, and gCC, connecting the anterior and medial temporal lobe to the prefrontal and orbitofrontal cortex.^43^ These tracts have been previously reported to be abnormal in cohorts of symptomatic mutation carriers,^3,44^ but not presymptomatically.^40^ The data of GM and WM differences in *MAPT* mutation carriers seems to suggest that abnormalities might come first in the anterior-medial temporal regions, with further spread not long before symptom onset via structural connectivity to the rest of the frontal and limbic areas, but multimodal longitudinal studies on large cohorts are needed to investigate this further.

*GRN* mutation carriers showed minor abnormalities at the presymptomatic stages, both in the GM (presubiculum, cerebellum and temporal pole) and WM (sCR, aCR, and UF), in line with existing literature.^3,7,45^ However, at the symptomatic stage the abnormalities were severe and widespread to cortical and subcortical regions, with all WM tracts severely abnormal.^2,3,46,47^

Overall, abnormalities in GM and WM regions seem to suggest that the brain is affected extremely early in *C9orf72* expansion carriers, presents early localized abnormalities in *MAPT* mutation carriers, and only shows changes at a later stage in *GRN* mutation carriers. The presence of abnormalities in *MAPT* and *GRN* mutation carriers closer to symptom onset is also reported by a longitudinal multimodal study.^48^ The relationship between WM and GM changes detectable *in vivo* on MRI and the underlining pathological changes in the three genetic groups is still to be fully understood, especially considering the heterogeneity within the same genetic group. According to the ‘molecular nexopathy’ paradigm,^49^ within affected brain networks there could be preferentially vulnerable hubs that different abnormal proteins (tau in *MAPT*, TDP-43 in *GRN* and *C9orf72*, with additional dipeptide repeat proteins in the latter) can differentially target and damage, leading to diverse symptoms and disease progression.

Across all the three genetic groups, presymptomatic carriers with normal *w*-scores for brain regions at baseline did not show large progression in their average clinical, cognitive, or behavioral scores after 12 months. Even when there was a significant change over time (such as in *GRN* and *C9orf72*), this was less than one point at the CDR® plus NACC FTLD total score, and less than 2 points at the CBI-R total scores, both lower than the change in the abnormal groups. The only exception was the MD for the sCC in the *C9orf72* expansion carriers, showing an increase in 3 points at the CDR® plus NACC FTLD total score, similar to the magnitude of change in the abnormal groups. This result might suggest that the clinical progression in presymptomatic *C9orf72* expansion carriers is not related to diffusion measures in sCC, but this has to be confirmed in further cohorts.

In contrast, presymptomatic mutation carriers with regional brain *w*-scores below the 10th percentile of the controls had significantly worse scores on average after one year. Abnormality on diffusion measures seem to lead to slightly larger significant differences in progression than abnormality in GM volumes, at least for *MAPT* and *C9orf72* mutation carriers for the behavioral scores, whilst the extent of progression was similar between GM and WM regions for *GRN* mutation carriers. One explanation could be that the GM atrophy is slower than WM diffusivity. One study has reported a significant longitudinal rate of change in WM for *MAPT* presymptomatic mutation carriers but not for *C9orf72* and *GRN* mutation carriers.^40^

The results of this study are particularly important when defining biomarkers to stratify participants in future trials. By only using total brain volume, one can predict if *GRN* presymptomatic mutation carriers are likely to progress 3 points on the CDR® plus NACC-FTLD sum of boxes score in 12 months. The whole brain volume was not as informative in *C9orf72* expansion carriers, with progression of only 1 point, but this is not surprising probably due to the slow progression of this genetic form, as reported by Staffaroni *et al*.^50^ Due to the small number in the subgroups who had abnormal whole brain volume at baseline and available follow-up data, unfortunately the results were inconclusive for *MAPT* mutation carriers and for progression on the CBI-R total score in all three groups. There could be the potential of an 11–16 points increase on the CBI-R total score in *MAPT* and *GRN* presymptomatic mutation carriers with abnormal whole brain volume, but larger studies are needed to confirm this.

However, specific regional measures for each of the genetic forms are associated with a larger increase in clinical scores. For *C9orf72* and *GRN* mutation carriers, both GM and WM tracts were associated with a similar worsening in behavioral symptoms, with a maximum of 8–10 points. *MAPT* mutation carriers showed a maximum of 11 points which showed the potential of being higher in a number of regions (up to 19 points) if this could be confirmed in larger samples. However, in contrast, for the CDR® plus NACC-FTLD sum of boxes, both *GRN* and *C9orf72* mutation carriers showed a larger increase compared to *MAPT* mutation carriers (4–5 points versus 2 points, which were not statistically significant). This may be related to differences in the types of clinical features detected by the CDR® plus NACC-FTLD in comparison to the CBI-R, with more cognitive and linguistic aspects that are seen in *GRN* and *C9orf72* mutation carriers measured by the CDR.

Despite abnormality on diffusion indexes seeming to be associated with slightly larger changes in clinical scores, abnormality in GM volumes was still associated with a significant change in both the scales used. This is particularly important, as diffusion measures are usually more difficult to obtain than volumes because of higher scanner requirements to acquire the sequences, measurement variability across different scanner types, and the advanced processing required to extract the measures. In addition, diffusion imaging is more prone to image artifacts than conventional T1-weighted structural imaging.

Future studies need to clarify what is a clinically relevant change in such clinical scores, and if the increase predicted by GM volumes are sufficient to discriminate ‘progressors’ versus ‘non progressors’ in the context of clinical trials. Moreover, it will be important to analyse the longitudinal evolution of brain changes and their correlations with the development and onset of symptoms. Another important future investigation is the detailed analyses of which cognitive or behavioral change would be better predicted by abnormal brain features at baseline, and how other variables can contribute to these different profiles of progression. In this study, we only focused on the global scores, but a dedicated investigation of the single subscores and specific cognitive domains is needed, including also measures that might predict motor phenotypes, especially for *C9orf72* expansion carriers. Moreover, as these findings are derived from group-level analyses, their relevance and application at the level of the single individual has still to be demonstrated.

This study has some limitations, including the difficulty of investigating small brain nuclei and tracts, which can be only accurately measured with the higher spatial resolution and contrast provided by high field MRI. For this reason, when looking at the progression of clinical and behavioral scores, we only focused on the whole structures. Moreover, the MR images were acquired from different scanners: despite the correction for manufacturer, together with other confounding variables, when computing the *w*-scores, we cannot assume their effects have been fully excluded. Moreover, in subgroups with less than 6 cases, the limitations of the Wilcoxon Signed Rank test meant that despite showing large changes these could not reach statistical significance. Further studies with larger samples are important to provide evidence on this matter.

Another important study would be to investigate the threshold for abnormality of *w*-scores by setting the threshold at the 5th percentile, rather than the 10th, to determine if even larger differences are seen over time in these more stringent subgroups. We examined this threshold in the current cohort, but, unfortunately, the small sample size of the abnormal subgroups prevented further analysis from being possible, and larger samples are needed.

In summary, by looking at *in vivo* regional volumetry, we have quantified and localized regional abnormalities on MRI in presymptomatic and symptomatic mutation carriers, and were able to detect different profiles of clinical and behavioral changes over time from brain abnormalities at baseline. This provides important evidence that imaging biomarkers can be helpful in designing clinical trials at the presymptomatic stages of genetic FTD.

## Supplementary Material

fcad061_Supplementary_Data

## APPENDIX

### List of GENFI consortium authors

Aitana Sogorb Esteve, Annabel Nelson, Carolin Heller, Caroline V. Greaves, Hanya Benotmane, Henrik Zetterberg, Imogen J. Swift, Kiran Samra, Rachelle Shafei, Carolyn Timberlake, Thomas Cope, Timothy Rittman, Alberto Benussi, Enrico Premi, Roberto Gasparotti, Silvana Archetti, Stefano Gazzina, Valentina Cantoni, Andrea Arighi, Chiara Fenoglio, Elio Scarpini, Giorgio Fumagalli, Vittoria Borracci, Giacomina Rossi, Giorgio Giaccone, Giuseppe Di Fede, Paola Caroppo, Pietro Tiraboschi, Sara Prioni, Veronica Redaelli, David Tang-Wai, Ekaterina Rogaeva, Miguel Castelo-Branco, Morris Freedman, Ron Keren, Sandra Black, Sara Mitchell, Christen Shoesmith, Robart Bartha, Rosa Rademakers, Jackie Poos, Janne M. Papma, Lucia Giannini, Rick van Minkelen, Yolande Pijnenburg, Benedetta Nacmias, Camilla Ferrari, Cristina Polito, Gemma Lombardi, Valentina Bessi, Michele Veldsman, Christin Andersson, Hakan Thonberg, Linn Öijerstedt, Vesna Jelic, Paul Thompson, Tobias Langheinrich, Albert Lladó, Anna Antonell, Jaume Olives, Mircea Balasa, Nuria Bargalló, Sergi Borrego-Ecija, Ana Verdelho, Carolina Maruta, Catarina B. Ferreira, Gabriel Miltenberger, Frederico Simões do Couto, Alazne Gabilondo, Ana Gorostidi, Jorge Villanua, Marta Cañada, Mikel Tainta, Miren Zulaica, Myriam Barandiaran, Patricia Alves, Benjamin Bender, Carlo Wilke, Lisa Graf, Annick Vogels, Mathieu Vandenbulcke, Philip Van Damme, Rose Bruffaerts, Koen Poesen, Pedro Rosa-Neto, Serge Gauthier, Agnès Camuzat, Alexis Brice, Anne Bertrand, Aurélie Funkiewiez, Daisy Rinaldi, Dario Saracino, Olivier Colliot, Sabrina Sayah, Catharina Prix, Elisabeth Wlasich, Olivia Wagemann, Sandra Loosli, Sonja Schönecker, Tobias Hoegen, Jolina Lombardi, Sarah Anderl-Straub, Adeline Rollin, Gregory Kuchcinski, Maxime Bertoux, Thibaud Lebouvier, Vincent Deramecourt, Beatriz Santiago, Diana Duro, Maria João Leitão, Maria Rosario Almeida, Miguel Tábuas-Pereira, Sónia Afonso.

## Abbreviations

aCR =: anterior corona radiata
aIC =: anterior part of the internal capsule
ALS =: amyotrophic lateral sclerosis
bCC =: body of the corpus callosum
bvFTD =: behavioural variant of frontotemporal dementia
CBI-R =: revised version of the Cambridge Behavioural Inventory
CBS =: corticobasal syndrome
*C9orf72* =: chromosome 9 open reading frame 72
DLPFC =: dorsolateral prefrontal cortex
EC =: external capsule
FA =: fractional anisotropy
FTD =: frontotemporal dementia
gCC =: genu of the corpus callosum
GENFI =: GENetic Frontotemporal dementia Initiative
GIF =: geodesic information flow
GM =: grey matter
*GRN* =: progranulin
JHU =: John Hopkins University
i-tub =: inferior tuberal
LD =: laterodorsal
LGN =: lateral geniculate nucleus
*MAPT* =: microtubule-associated protein tau
MD =: mean diffusivity
MED PARIETAL =: medial parietal
MGN =: medial geniculate nucleus
N/A =: not applicable
N/I =: not included in the analyses
NOS =: not otherwise specified
pCR =: posterior corona radiata
pIC =: posterior part of the internal capsule
PPA =: primary progressive aphasia
PSP =: progressive supranuclear palsy
pTR =: posterior thalamic radiation
rIC =: retrolenticular part of the internal capsule
ROI =: region of interest
sCC =: splenium of the corpus callosum
sCR =: superior corona radiata
SD =: standard deviation
SLF =: superior longitudinal fasciculus
SS =: sagittal stratum
s-tub =: superior tuberal
TIV =: total intracranial volume
UF =: uncinate fasciculus
VMPFC =: ventromedial prefrontal cortex
WM =: white matter
