# Supplementary material for: Structural MRI predicts clinical progression in presymptomatic genetic frontotemporal dementia: findings from the GENetic Frontotemporal dementia Initiative cohort
Source: Brain Commun. 2023 Mar 10;5(2):fcad061. doi: 10.1093/braincomms/fcad061 (PMC10036293; doi:10.1093/braincomms/fcad061)
Supplement: fcad061_Supplementary_Data [file fcad061_supplementary_data.zip › Supplementary_material.docx]

**Supplementary table legends**

In case of formatting issues, the Supplementary Tables can be downloaded here: www.dropbox.com/s/40wjebpxpc4h331/Bocchetta_Suppl_Tables.xlsx?dl=0

**Supplementary Table 1. Overview of MRI scanners and acquisition parameters**. Abbreviations: GE General Electric; FOV field of view; TI inversion time; TR repetition time; TE echo time; FM field map; EPI echo-planar imaging; PE phase-encoding; AP anterior-posterior.

**Supplementary Table 2:** **Grey matter regions for *C9orf72*, *MAPT* and *GRN* for the stages defined by CDR®+NACC FTLD global scores.** Values denote mean and standard deviation (SD) for the w-scores. Bold indicates significantly negative w-scores after Bonferroni correction for multiple comparisons. Abbreviations. Cortex: DLPFC dorsolateral prefrontal, VMPFC ventromedial prefrontal; Brainstem and Cerebellum: SCP superior cerebellar peduncle; VIIA – CI lobule VIIA – Crus I, VIIA – CII lobule VIIA – Crus II, DN deep nuclei; Amygdala: CAT cortico-amygdaloid transition area, Sup superficial nuclei, AB accessory basal nucleus; Hippocampus: DG dentate gyrus, CA cornu ammonis; Thalamus: AV anteroventral, VA ventral anterior, LD laterodorsal, VLa ventral lateral anterior, MD mediodorsal, LP lateral posterior, VLp ventral lateral posterior, VPL ventral posterolateral, VM ventromedial, LGN lateral geniculate nucleus, MGN medial geniculate nucleus; Hypothalamus: as anterior superior, ai anterior inferior, s-tub superior tuberal, i-tub inferior tuberal.

**Supplementary Table 3:** **White matter regions for *C9orf72*, *MAPT* and *GRN* for the stages defined by CDR®+NACC FTLD global scores.** Values denote mean and standard deviation (SD) for the w-scores in the fractional anisotropy (FA) and mean diffusivity (MD). Bold indicates significantly negative w-scores after Bonferroni correction for multiple comparisons. Abbreviations. UF uncinate fasciculus, SLF superior longitudinal fasciculus, SS sagittal stratum, pTR posterior thalamic radiation, aCR anterior corona radiata, pCR posterior corona radiata, sCR superior corona radiata, EC external capsule, aIC anterior part of the internal capsule, pIC posterior part of the internal capsule, rIC retrolenticular part of the internal capsule, gCC genu of the corpus callosum, bCC body of the corpus callosum, sCC splenium of the corpus callosum.

**Supplementary Table 4:** **Longitudinal progression at the CDR®+NACC FTLD sum of boxes scores for the “normal” and “abnormal” groups for each of the ROIs in the *C9orf72*, *MAPT* and *GRN* presymptomatic carriers.** Red indicates significantly difference in time. Abbreviations. FA fractional anisotropy, MD mean diffusivity, SD standard deviation, Diff mean difference between the two visits, 95% CI 95% confidence interval of the mean difference, DLPFC dorsolateral prefrontal, VMPFC ventromedial prefrontal, UF uncinate fasciculus, SLF superior longitudinal fasciculus, SS sagittal stratum, pTR posterior thalamic radiation, aCR anterior corona radiata, pCR posterior corona radiata, sCR superior corona radiata, EC external capsule, aIC anterior part of the internal capsule, pIC posterior part of the internal capsule, rIC retrolenticular part of the internal capsule, gCC genu of the corpus callosum, bCC body of the corpus callosum, sCC splenium of the corpus callosum.

**Supplementary Table 5:** **Longitudinal progression at the CBI-R total scores for the “normal” and “abnormal” groups for each of the ROIs in the *C9orf72*, *MAPT* and *GRN* presymptomatic carriers.** Red indicates significantly difference in time. Abbreviations. FA fractional anisotropy, MD mean diffusivity, SD standard deviation, Diff mean difference between the two visits, 95% CI 95% confidence interval of the mean difference, DLPFC dorsolateral prefrontal, VMPFC ventromedial prefrontal, UF uncinate fasciculus, SLF superior longitudinal fasciculus, SS sagittal stratum, pTR posterior thalamic radiation, aCR anterior corona radiata, pCR posterior corona radiata, sCR superior corona radiata, EC external capsule, aIC anterior part of the internal capsule, pIC posterior part of the internal capsule, rIC retrolenticular part of the internal capsule, gCC genu of the corpus callosum, bCC body of the corpus callosum, sCC splenium of the corpus callosum.

**Supplementary Material:** Detailed description of the results from the GM and WM analyses at baseline.

***Baseline GM volumes***

At the presymptomatic stage, *C9orf72* expansion carriers had significantly lower w-scores in all the cortical regions except for the VMPFC, medial temporal and orbitofrontal cortex (**Figure 2**, **Supplementary Table 2**). Among the subcortical regions, the putamen and globus pallidus had significantly lower volumes, together with the lobule V, VI, VIIa-Crus II, VIIb and VIIIb of the cerebellum. The amygdala and hippocampus also had significantly lower volumes (except for the hippocampal tail and subiculum), as did the s-tub region of the hypothalamus. The thalamus was the structure with the most abnormal regions: the pulvinar had values below the 10^th^ percentile of the controls, and the mediodorsal, anteroventral, ventral anterior, ventral lateral anterior, lateral posterior and LGN had values below the 25^th^ percentile (**Figure 2**, **Supplementary Table 2**). Only the ventromedial thalamic region was not significantly smaller than controls. At the fully symptomatic stage, *C9orf72* expansion carriers had significantly lower w-scores in all the cortical regions, with values below the 2.5^th^ percentile in the insula and dorsolateral temporal cortex (**Figure 2**, **Supplementary Table 2**), and below the 5^th^ percentile in the DLPFC, motor and lateral parietal, and below the 10^th^ percentile in the orbitofrontal cortex. The putamen and globus pallidus had values <5^th^ and <10^th^ percentile respectively, and the cerebellum had significantly lower values (<25^th^ percentile) in the VIIIa and VIIIb, and in the I-IV, VI, VIIa-Crus II, VIIb and vermis (>25^th^ percentile). The amygdalar and hippocampal regions were all below the 2.5^th^ percentile, except for the lateral amygdala, presubiculum and subiculum (<5^th^ percentile) and hippocampal tail (<10^th^ percentile). The hypothalamus had values <10^th^ percentile in the anterior-superior, s-tub and posterior, and <25^th^ percentile in the anterior-inferior. Finally, all the thalamic regions were affected, with values <2.5^th^ percentile in the mediodorsal and anteroventral, <5^th^ percentile in the lateral posterior and pulvinar, <10^th^ percentile in the midline, intralaminar and LD, <25^th^ percentile in the ventral anterior, ventral lateral anterior, ventral lateral posterior and LGN.

At the presymptomatic stage, *MAPT* mutation carriers had significantly lower w-scores in the dorsolateral temporal cortex, but not in other cortical, cerebellar or brainstem regions (**Figure 2**, **Supplementary Table 2**). All regions of the amygdala were significantly lower than controls (except for the lateral region), with the accessory basal nucleus showing values <25^th^ percentile. The subiculum and presubiculum were also significantly lower, together with the LGN in the thalamus. When fully symptomatic, *MAPT* mutation carriers showed extremely low values (<2.5^th^ percentile) in all the temporal cortex, amygdala, hippocampus and insula. The cingulate (<25^th^ percentile) and orbitofrontal cortex were also significantly lower (**Figure 2**, **Supplementary Table 2**). The values of the nucleus accumbens, globus pallidus, and midbrain were all below the 25^th^ percentile, while the putamen values were below the 10^th^. The hypothalamus showed values <2.5^th^ percentile in the anterior-superior, s-tub and posterior, <10^th^ in the anterior-inferior, and <25^th^ in the i-tub. All the thalamic regions (except for the LGN) were significantly lower than the 25^th^ percentile, with the mediodorsal values <5^th^ and the anteroventral values <10^th^ percentile.

Presymptomatic *GRN* mutation carriers showed significantly lower values in the temporal pole, presubiculum, and in the anterior superior cerebellum (lobule I-IV, V and VI). No other brain region was affected in this early stage (**Figure 2**, **Supplementary Table 2**). Fully symptomatic *GRN* mutation carriers showed significantly lower volumes in all cortical regions, except for the sensory cortex, with extremely low w-scores (<2.5^th^ percentile) in the DLPFC, insula and motor cortex, values <5^th^ percentile in the dorsolateral temporal and lateral parietal cortex, <10^th^ in the cingulate and orbitofrontal cortex, and <25^th^ in the VMPFC, temporal pole, medial parietal and occipital cortex. The w-scores of the globus pallidus and the putamen were below the 25^th^ and 5^th^ percentile respectively, while among the brainstem and cerebellar regions, the midbrain and lobule VIIb were below the 25^th^ percentile, while the pons, lobule VIIa-Crus I, VIIa-Crus II, VIIIa and VIIIb were also significantly lower than control distribution. All amygdalar and hippocampal regions were significantly lower than the 25^th^ percentile, with the lowest values in the presubiculum (<2.5^th^), accessory basal nucleus (<5^th^), basal and paralaminar nucleus, cortico-amygdaloid transition area, superficial nuclei, CA1, CA2/CA3, CA4, dentate gyrus, and subiculum (<10^th^ percentile). All the thalamic regions (except for the LGN) were significantly lower than the controls, with the mediodorsal values <2.5^th^ percentile, the midline, anteroventral and ventral anterior values <5^th^ percentile, the lateral posterior and ventral lateral anterior values <10^th^ percentile, and LD, intralaminar, ventromedial, ventral posterolateral and ventral lateral posterior values <25^th^ percentile. The posterior hypothalamus showed values <2.5^th^ percentile, the anterior-superior and s-tub <10^th^, and anterior-inferior <25^th^ percentile.

***Baseline diffusion WM indices***

Presymptomatic *C9orf72* expansion carriers showed FA values lower than controls in the SS, whole corpus callosum, pTR, aCR, EC, and aIC (**Figure 3**, **Supplementary Table 3**). MD values were higher than controls in the SS, gCC and sCC, pTR, aCR, pCR, EC, cingulum, and SLF. Symptomatic *C9orf72* expansion carriers showed FA values lower than controls in all WM tracts except the sCR (not significant), with particularly lower values in the gCC and aCR (<2.5^th^ percentile), aIC, SS, and bCC (<5^th^ percentile), UF, sCC and cingulum (<10^th^ percentile). MD values were <10^th^ percentile in all tracts, expect for the pIC (<25^th^ percentile), with particularly abnormal values (<2.5^th^ percentile) in the SS, corpus callosum (genu and body), aCR, sCR, cingulum, pTR, and aIC (**Figure 3**, **Supplementary Table 3**).

Presymptomatic *MAPT* mutation carriers only showed significantly lower FA than controls in the aIC (**Figure 3**, **Supplementary Table 3**). Once symptoms were present, *MAPT* mutation carriers showed FA values <2.5^th^ percentile for the UF, <10^th^ percentile in the gCC and cingulum, <25^th^ percentile in the SS, aCR and SLF, and significantly lower values in the pTR. MD values were significantly <2.5^th^ percentile of controls in the UF and SS, <5^th^ percentile in the aCR and <10^th^ in the gCC.

At a presymptomatic stage, *GRN* mutation carriers showed significantly lower FA than controls in the sCR, and significantly higher MD than controls in the UF and aCR (**Figure 3**, **Supplementary Table 3**). Fully symptomatic *GRN* mutation carriers showed abnormal FA and MD values in all tracts (**Figure 3**, **Supplementary Table 3**). FA values were <2.5^th^ percentile in the corpus callosum (genu and body), cingulum, aIC, and aCR; <5^th^ percentile in the UF, SS, EC, SLF and sCC. Interestingly, nearly all tracts showed MD values <2.5^th^ percentile, except for the sCC (<5^th^ percentile), UF (<10^th^ percentile), pIC and rIC (<25^th^ percentile) (**Figure 3**, **Supplementary Table 3**).

**Appendix**

***List of GENFI consortium authors:***

| **Author** | **Affiliation** |
| --- | --- |
| Aitana Sogorb Esteve | Department of Neurodegenerative Disease, Dementia Research Centre, UCL Queen Square Institute of Neurology, London, UK;  UK Dementia Research Institute at University College London, UCL Queen Square Institute of Neurology, London, UK |
| Annabel Nelson | Department of Neurodegenerative Disease, Dementia Research Centre, UCL Queen Square Institute of Neurology, London, UK |
| Carolin Heller | Department of Neurodegenerative Disease, Dementia Research Centre, UCL Queen Square Institute of Neurology, London, UK |
| Caroline V. Greaves | Department of Neurodegenerative Disease, Dementia Research Centre, UCL Queen Square Institute of Neurology, London, UK |
| Hanya Benotmane | UK Dementia Research Institute at University College London, UCL Queen Square Institute of Neurology, London, UK |
| Henrik Zetterberg | UK Dementia Research Institute at University College London, UCL Queen Square Institute of Neurology, London, UK; Department of Psychiatry and Neurochemistry, the Sahlgrenska Academy at the University of Gothenburg, Mölndal, Sweden |
| Imogen J Swift | Department of Neurodegenerative Disease, Dementia Research Centre, UCL Queen Square Institute of Neurology, London, UK; UK Dementia Research Institute at University College London, UCL Queen Square Institute of Neurology, London, UK |
| Kiran Samra | Department of Neurodegenerative Disease, Dementia Research Centre, UCL Queen Square Institute of Neurology, London, UK |
| Rachelle Shafei | Department of Neurodegenerative Disease, Dementia Research Centre, UCL Queen Square Institute of Neurology, London, UK |
| Carolyn Timberlake | Department of Clinical Neurosciences, University of Cambridge, Cambridge, UK |
| Thomas Cope | Department of Clinical Neuroscience, University of Cambridge, Cambridge, UK |
| Timothy Rittman | Department of Clinical Neurosciences, University of Cambridge, Cambridge, UK |
| Alberto Benussi | Centre for Neurodegenerative Disorders, Department of Clinical and Experimental Sciences, University of Brescia, Brescia, Italy |
| Enrico Premi | Stroke Unit, ASST Brescia Hospital, Brescia, Italy |
| Roberto Gasparotti | Neuroradiology Unit, University of Brescia, Brescia, Italy |
| Silvana Archetti | Biotechnology Laboratory, Department of Diagnostics, ASST Brescia Hospital, Brescia, Italy |
| Stefano Gazzina | Neurology, ASST Brescia Hospital, Brescia, Italy |
| Valentina Cantoni | Centre for Neurodegenerative Disorders, Department of Clinical and Experimental Sciences, University of Brescia, Brescia, Italy |
| Andrea Arighi | Fondazione IRCCS Ca’ Granda Ospedale Maggiore Policlinico, Neurodegenerative Diseases Unit, Milan, Italy; University of Milan, Centro Dino Ferrari, Milan, Italy |
| Chiara Fenoglio | Fondazione IRCCS Ca’ Granda Ospedale Maggiore Policlinico, Neurodegenerative Diseases Unit, Milan, Italy; University of Milan, Centro Dino Ferrari, Milan, Italy |
| Elio Scarpini | Fondazione IRCCS Ca’ Granda Ospedale Maggiore Policlinico, Neurodegenerative Diseases Unit, Milan, Italy; University of Milan, Centro Dino Ferrari, Milan, Italy |
| Giorgio Fumagalli | Fondazione IRCCS Ca’ Granda Ospedale Maggiore Policlinico, Neurodegenerative Diseases Unit, Milan, Italy; University of Milan, Centro Dino Ferrari, Milan, Italy |
| Vittoria Borracci | Fondazione IRCCS Istituto Neurologico Carlo Besta, Milano, Italy |
| Giacomina Rossi | Fondazione IRCCS Istituto Neurologico Carlo Besta, Milano, Italy |
| Giorgio Giaccone | Fondazione IRCCS Istituto Neurologico Carlo Besta, Milano, Italy |
| Giuseppe Di Fede | Fondazione IRCCS Istituto Neurologico Carlo Besta, Milano, Italy |
| Paola Caroppo | Fondazione IRCCS Istituto Neurologico Carlo Besta, Milano, Italy |
| Pietro Tiraboschi | Fondazione IRCCS Istituto Neurologico Carlo Besta, Milano, Italy |
| Sara Prioni | Fondazione IRCCS Istituto Neurologico Carlo Besta, Milano, Italy |
| Veronica Redaelli | Fondazione IRCCS Istituto Neurologico Carlo Besta, Milano, Italy |
| David Tang-Wai | The University Health Network, Krembil Research Institute, Toronto, Canada |
| Ekaterina Rogaeva | Tanz Centre for Research in Neurodegenerative Diseases, University of Toronto, Toronto, Canada |
| Miguel Castelo-Branco | Faculty of Medicine, University of Coimbra, Coimbra, Portugal |
| Morris Freedman | Baycrest Health Sciences, Rotman Research Institute, University of Toronto, Toronto, Canada |
| Ron Keren | The University Health Network, Toronto Rehabilitation Institute, Toronto, Canada |
| Sandra Black | Sunnybrook Health Sciences Centre, Sunnybrook Research Institute, University of Toronto, Toronto, Canada |
| Sara Mitchell | Sunnybrook Health Sciences Centre, Sunnybrook Research Institute, University of Toronto, Toronto, Canada |
| Christen Shoesmith | Department of Clinical Neurological Sciences, University of Western Ontario, London, Ontario, Canada |
| Robart Bartha | Department of Medical Biophysics, The University of Western Ontario, London, Ontario, Canada; Centre for Functional and Metabolic Mapping, Robarts Research Institute, The University of Western Ontario, London, Ontario, Canada |
| Rosa Rademakers | Center for Molecular Neurology, University of Antwerp |
| Jackie Poos | Department of Neurology, Erasmus Medical Center, Rotterdam, Netherlands |
| Janne M. Papma | Department of Neurology, Erasmus Medical Center, Rotterdam, Netherlands |
| Lucia Giannini | Department of Neurology, Erasmus Medical Center, Rotterdam, Netherlands |
| Rick van Minkelen | Department of Clinical Genetics, Erasmus Medical Center, Rotterdam, Netherlands |
| Yolande Pijnenburg | Amsterdam University Medical Centre, Amsterdam Vumc, Amsterdam, Netherlands |
| Benedetta Nacmias | Department of Neuroscience, Psychology, Drug Research and Child Health, University of Florence, Florence, Italy |
| Camilla Ferrari | Department of Neuroscience, Psychology, Drug Research and Child Health, University of Florence, Florence, Italy |
| Cristina Polito | Department of Biomedical, Experimental and Clinical Sciences “Mario Serio”, Nuclear Medicine Unit, University of Florence, Florence, Italy |
| Gemma Lombardi | Department of Neuroscience, Psychology, Drug Research and Child Health, University of Florence, Florence, Italy |
| Valentina Bessi | Department of Neuroscience, Psychology, Drug Research and Child Health, University of Florence, Florence, Italy |
| Michele Veldsman | Nuffield Department of Clinical Neurosciences, Medical Sciences Division, University of Oxford, Oxford, UK |
| Christin Andersson | Department of Clinical Neuroscience, Karolinska Institutet, Stockholm, Sweden |
| Hakan Thonberg | Center for Alzheimer Research, Division of Neurogeriatrics, Karolinska Institutet, Stockholm, Sweden |
| Linn Öijerstedt | Center for Alzheimer Research, Division of Neurogeriatrics, Department of Neurobiology, Care Sciences and Society, Bioclinicum, Karolinska Institutet, Solna, Sweden; Unit for Hereditary Dementias, Theme Aging, Karolinska University Hospital, Solna, Sweden |
| Vesna Jelic | Division of Clinical Geriatrics, Karolinska Institutet, Stockholm, Sweden |
| Paul Thompson | Division of Neuroscience and Experimental Psychology, Wolfson Molecular Imaging Centre, University of Manchester, Manchester, UK |
| Tobias Langheinrich | Division of Neuroscience and Experimental Psychology, Wolfson Molecular Imaging Centre, University of Manchester, Manchester, UK; Manchester Centre for Clinical Neurosciences, Department of Neurology, Salford Royal NHS Foundation Trust, Manchester, UK |
| Albert Lladó | Alzheimer’s disease and Other Cognitive Disorders Unit, Neurology Service, Hospital Clínic, Barcelona, Spain |
| Anna Antonell | Alzheimer’s disease and Other Cognitive Disorders Unit, Neurology Service, Hospital Clínic, Barcelona, Spain |
| Jaume Olives | Alzheimer’s disease and Other Cognitive Disorders Unit, Neurology Service, Hospital Clínic, Barcelona, Spain |
| Mircea Balasa | Alzheimer’s disease and Other Cognitive Disorders Unit, Neurology Service, Hospital Clínic, Barcelona, Spain |
| Nuria Bargalló | Imaging Diagnostic Center, Hospital Clínic, Barcelona, Spain |
| Sergi Borrego-Ecija | Alzheimer’s disease and Other Cognitive Disorders Unit, Neurology Service, Hospital Clínic, Barcelona, Spain |
| Ana Verdelho | Department of Neurosciences and Mental Health, Centro Hospitalar Lisboa Norte - Hospital de Santa Maria & Faculty of Medicine, University of Lisbon, Lisbon, Portugal |
| Carolina Maruta | Laboratory of Language Research, Centro de Estudos Egas Moniz, Faculty of Medicine, University of Lisbon, Lisbon, Portugal |
| Catarina B. Ferreira | Laboratory of Neurosciences, Faculty of Medicine, University of Lisbon, Lisbon, Portugal |
| Gabriel Miltenberger | Faculty of Medicine, University of Lisbon, Lisbon, Portugal |
| Frederico Simões do Couto | Faculdade de Medicina, Universidade Católica Portuguesa |
| Alazne Gabilondo | Cognitive Disorders Unit, Department of Neurology, Donostia University Hospital, San Sebastian, Gipuzkoa, Spain; Neuroscience Area, Biodonostia Health Research Insitute, San Sebastian, Gipuzkoa, Spain |
| Ana Gorostidi | Neuroscience Area, Biodonostia Health Research Institute, San Sebastian, Gipuzkoa, Spain |
| Jorge Villanua | OSATEK, University of Donostia, San Sebastian, Gipuzkoa, Spain |
| Marta Cañada | CITA Alzheimer, San Sebastian, Gipuzkoa, Spain |
| Mikel Tainta | Neuroscience Area, Biodonostia Health Research Insitute, San Sebastian, Gipuzkoa, Spain |
| Miren Zulaica | Neuroscience Area, Biodonostia Health Research Insitute, San Sebastian, Gipuzkoa, Spain |
| Myriam Barandiaran | Cognitive Disorders Unit, Department of Neurology, Donostia University Hospital, San Sebastian, Gipuzkoa, Spain; Neuroscience Area, Biodonostia Health Research Insitute, San Sebastian, Gipuzkoa, Spain |
| Patricia Alves | Neuroscience Area, Biodonostia Health Research Insitute, San Sebastian, Gipuzkoa, Spain; Department of Educational Psychology and Psychobiology, Faculty of Education, International University of La Rioja, Logroño, Spain |
| Benjamin Bender | Department of Diagnostic and Interventional Neuroradiology, University of Tübingen, Tübingen, Germany |
| Carlo Wilke | Department of Neurodegenerative Diseases, Hertie-Institute for Clinical Brain Research and Center of Neurology, University of Tübingen, Tübingen, Germany; Center for Neurodegenerative Diseases (DZNE), Tübingen, Germany |
| Lisa Graf | Department of Neurodegenerative Diseases, Hertie-Institute for Clinical Brain Research and Center of Neurology, University of Tübingen, Tübingen, Germany |
| Annick Vogels | Department of Human Genetics, KU Leuven, Leuven, Belgium |
| Mathieu Vandenbulcke | Geriatric Psychiatry Service, University Hospitals Leuven, Belgium; Neuropsychiatry, Department of Neurosciences, KU Leuven, Leuven, Belgium |
| Philip Van Damme | Neurology Service, University Hospitals Leuven, Belgium; Laboratory for Neurobiology, VIB-KU Leuven Centre for Brain Research, Leuven, Belgium |
| Rose Bruffaerts | Department of Biomedical Sciences, University of Antwerp, Antwerp, Belgium; Biomedical Research Institute, Hasselt University, 3500 Hasselt, Belgium |
| Koen Poesen | Laboratory for Molecular Neurobiomarker Research, KU Leuven, Leuven, Belgium |
| Pedro Rosa-Neto | Translational Neuroimaging Laboratory, McGill Centre for Studies in Aging, McGill University, Montreal, Québec, Canada |
| Serge Gauthier | Alzheimer Disease Research Unit, McGill Centre for Studies in Aging, Department of Neurology & Neurosurgery, McGill University, Montreal, Québec, Canada |
| Agnès Camuzat | Sorbonne Université, Paris Brain Institute – Institut du Cerveau – ICM, Inserm U1127, CNRS UMR 7225, AP-HP - Hôpital Pitié-Salpêtrière, Paris, France |
| Alexis Brice | Sorbonne Université, Paris Brain Institute – Institut du Cerveau – ICM, Inserm U1127, CNRS UMR 7225, AP-HP - Hôpital Pitié-Salpêtrière, Paris, France; Reference Network for Rare Neurological Diseases (ERN-RND) |
| Anne Bertrand | Sorbonne Université, Paris Brain Institute – Institut du Cerveau – ICM, Inserm U1127, CNRS UMR 7225, AP-HP - Hôpital Pitié-Salpêtrière, Paris, France; Inria, Aramis project-team, F-75013, Paris, France; Centre pour l'Acquisition et le Traitement des Images, Institut du Cerveau et la Moelle, Paris, France |
| Aurélie Funkiewiez | Centre de référence des démences rares ou précoces, IM2A, Département de Neurologie, AP-HP - Hôpital Pitié-Salpêtrière, Paris, France; Sorbonne Université, Paris Brain Institute – Institut du Cerveau – ICM, Inserm U1127, CNRS UMR 7225, AP-HP - Hôpital Pitié-Salpêtrière, Paris, France |
| Daisy Rinaldi | Centre de référence des démences rares ou précoces, IM2A, Département de Neurologie, AP-HP - Hôpital Pitié-Salpêtrière, Paris, France; Sorbonne Université, Paris Brain Institute – Institut du Cerveau – ICM, Inserm U1127, CNRS UMR 7225, AP-HP - Hôpital Pitié-Salpêtrière, Paris, France; Département de Neurologie, AP-HP - Hôpital Pitié-Salpêtrière, Paris, France |
| Dario Saracino | Sorbonne Université, Paris Brain Institute – Institut du Cerveau – ICM, Inserm U1127, CNRS UMR 7225, AP-HP - Hôpital Pitié-Salpêtrière, Paris, France; Inria, Aramis project-team, F-75013, Paris, France; Centre de référence des démences rares ou précoces, IM2A, Département de Neurologie, AP-HP - Hôpital Pitié-Salpêtrière, Paris, France |
| Olivier Colliot | Sorbonne Université, Paris Brain Institute – Institut du Cerveau – ICM, Inserm U1127, CNRS UMR 7225, AP-HP - Hôpital Pitié-Salpêtrière, Paris, France; Inria, Aramis project-team, F-75013, Paris, France; Centre pour l'Acquisition et le Traitement des Images, Institut du Cerveau et la Moelle, Paris, France |
| Sabrina Sayah | Sorbonne Université, Paris Brain Institute – Institut du Cerveau – ICM, Inserm U1127, CNRS UMR 7225, AP-HP - Hôpital Pitié-Salpêtrière, Paris, France |
| Catharina Prix | Neurologische Klinik, Ludwig-Maximilians-Universität München, Munich, Germany |
| Elisabeth Wlasich | Neurologische Klinik, Ludwig-Maximilians-Universität München, Munich, Germany |
| Olivia Wagemann | Neurologische Klinik, Ludwig-Maximilians-Universität München, Munich, Germany |
| Sandra Loosli | Neurologische Klinik, Ludwig-Maximilians-Universität München, Munich, Germany |
| Sonja Schönecker | Neurologische Klinik, Ludwig-Maximilians-Universität München, Munich, Germany |
| Tobias Hoegen | Neurologische Klinik, Ludwig-Maximilians-Universität München, Munich, Germany |
| Jolina Lombardi | Department of Neurology, University of Ulm, Ulm |
| Sarah Anderl-Straub | Department of Neurology, University of Ulm, Ulm, Germany |
| Adeline Rollin | CHU, CNR-MAJ, Labex Distalz, LiCEND Lille, France |
| Gregory Kuchcinski | Univ Lille, France; Inserm 1172, Lille, France; CHU, CNR-MAJ, Labex Distalz, LiCEND Lille, France |
| Maxime Bertoux | Inserm 1172, Lille, France; CHU, CNR-MAJ, Labex Distalz, LiCEND Lille, France |
| Thibaud Lebouvier | Univ Lille, France; Inserm 1172, Lille, France; CHU, CNR-MAJ, Labex Distalz, LiCEND Lille, France |
| Vincent Deramecourt | Univ Lille, France; Inserm 1172, Lille, France; CHU, CNR-MAJ, Labex Distalz, LiCEND Lille, France |
| Beatriz Santiago | Neurology Department, Centro Hospitalar e Universitario de Coimbra, Coimbra, Portugal |
| Diana Duro | Faculty of Medicine, University of Coimbra, Coimbra, Portugal |
| Maria João Leitão | Centre of Neurosciences and Cell Biology, Universidade de Coimbra, Coimbra, Portugal |
| Maria Rosario Almeida | Faculty of Medicine, University of Coimbra, Coimbra, Portugal |
| Miguel Tábuas-Pereira | Neurology Department, Centro Hospitalar e Universitario de Coimbra, Coimbra, Portugal |
| Sónia Afonso | Instituto Ciencias Nucleares Aplicadas a Saude, Universidade de Coimbra, Coimbra, Portugal |
